# UNC5C Receptor Proteolytic Cleavage by Active AEP Promotes Dopaminergic Neuronal Degeneration in Parkinson's Disease

**DOI:** 10.1002/advs.202103396

**Published:** 2022-01-12

**Authors:** Guiqin Chen, Eun Hee Ahn, Seong Su Kang, Yiyuan Xia, Xia Liu, Zhaohui Zhang, Keqiang Ye

**Affiliations:** ^1^ Department of Pathology and Laboratory Medicine Emory University School of Medicine Atlanta GA 30322 USA; ^2^ Department of Neurology Renmin Hospital of Wuhan University Wuhan Hubei Province 430060 China; ^3^ Faculty of Life and Health Sciences Shenzhen Institute of Advanced Technology Chinese Academy of Science Shenzhen Guangdong 518035 China; ^4^ The Brain Cognition and Brain Disease Institute (BCBDI) Shenzhen Institute of Advanced Technology Chinese Academy of Science Shenzhen Guangdong 518035 China

**Keywords:** alpha‐synuclein, asparagine endopeptidase, netrin‐1, Parkinson's disease, UNC5C

## Abstract

Netrin‐1 is a chemotropic cue mediating axon growth and neural migration in neuronal development, and its receptors deletion in colorectal cancer and UNC5s act as dependence receptors regulating neuronal apoptosis. Asparagine endopeptidase (AEP) is an age‐dependent protease that cuts human alpha‐synuclein (*α*‐Syn) at N103 and triggers its aggregation and neurotoxicity. In the current study, it is reported that UNC5C receptor is cleaved by AEP in Parkinson's disease (PD) and facilitates dopaminergic neuronal loss. UNC5C is truncated by active AEP in human *α*‐SNCA transgenic mice in an age‐dependent manner or induced by neurotoxin rotenone. Moreover, UNC5C is fragmented by AEP in PD brains, inversely correlated with reduced netrin‐1 levels. Netrin‐1 deprivation in primary cultures induces AEP and caspase‐3 activation, triggering UNC5C proteolytic fragmentation and enhancing neuronal loss. Noticeably, blocking UNC5C cleavage by AEP attenuates netrin‐1 deprivation‐elicited neuronal death and motor disorders in netrin flox/flox mice. Overexpression of AEP‐truncated UNC5C intracellular fragment strongly elicits *α*‐Syn aggregation and dopaminergic loss, locomotor deficits in *α*‐SNCA transgenic mice. Hence, the findings demonstrate that netrin‐1 reduction and UNC5C truncation by AEP contribute to PD pathogenesis.

## Graphical abstract

Netrin‐1 is reduced in the brains of Parkinson's disease (PD) patients and PD genetic and neurotoxin mouse models, accompanied with prominent asparagine endopeptidase (AEP) activation and UNC5C fragmentation. Netrin‐1 deprivation in primary cultures induces AEP and caspase‐3 activation, triggering UNC5C fragmentation and neuronal loss. Overexpression of AEP‐truncated UNC5C fragments promotes alpha‐synuclein aggregation and dopaminergic neuronal loss in SNCA transgenic mice.

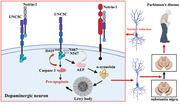

## 1 Introduction

Parkinson's disease (PD) is the second most common neurodegenerative disease with aging.^[^ ^1^ ^]^ PD is a progressive nervous system disorder that affects movement, and the motor symptoms include bradykinesia, tremor, rigidity, and later postural instability.^[^ ^2^ ^]^ The pathological hallmarks are characterized by the progressive degeneration of the dopaminergic nigrostriatal pathway, and the presence of Lewy bodies in the remnant dopaminergic neurons.^[^ ^3^ ^]^ The main component in Lewy bodies is aggregated and highly phosphorylated alpha‐synuclein (*α*‐Syn), an intracellular presynaptic protein.^[^ ^4^ ^]^ The etiology of PD appears to be multifactorial, involving both genetic and environmental components. Mounting evidence supports aggregated inclusions of *α*‐Syn, dysfunction of protein turnover and mitochondrial dysfunction as important mediators in the pathogenesis of PD, triggering prominent death of dopaminergic neurons in the substantia nigra pars compacta (SNpc).^[^ ^5^ ^]^ Recently, we showed that asparagine endopeptidase (AEP) cleaves human *α*‐Syn, triggers its aggregation and escalates its neurotoxicity, leading to dopaminergic neuronal loss and motor impairments in a mouse model. AEP is activated and cleaves human *α*‐Syn at N103 in an age‐dependent manner, and AEP is highly active in the SNpc regions in human brains with PD. Deletion of AEP from human *α*‐SNCA transgenic mice alleviates dopaminergic neuronal loss and diminishes motor deficits.^[^ ^6^ ^]^ In addition to *α*‐Syn, we have reported that AEP acts as *δ*‐secretase that truncates both APP N585 and Tau N368 in Alzheimer's disease (AD), promoting AD pathogenesis.^[^ ^7^ ^]^ Most recently, we report that AEP cleaves both *α*‐Syn at N103 and Tau at N368 in the enteric nervous system (ENS), and mediates their fibrillization and retrograde propagation from the gut to the brain, triggering nigra dopaminergic neuronal death associated with Lewy bodies and motor dysfunction. Hence, AEP plays a crucial role in initiating PD pathology progression from the ENS to the central nervous system (CNS).^[^ ^8^ ^]^

Netrin‐1 is a member of a large family of laminin‐related factors.^[^ ^9^ ^]^ Netrin‐1 activates intracellular signal transduction pathways via multiple receptors, including deletion in colorectal cancer (DCC) and UNC5 homolog family members (UNC5A‐D) in humans.^[^ ^10^ ^]^ Both netrin‐1 and DCC are expressed in the adult CNS, particularly in the substantia nigra and DCC is specifically expressed in dopaminergic neurons in the SNpc.^[^ ^11^ ^]^ Most recently, we report that silencing netrin‐1 in the adult substantia nigra of mice induces DCC cleavage and a significant loss of dopamine neurons, resulting in motor deficits. By contrast, overexpression of netrin‐1 or brain administration of recombinant netrin‐1 is neuroprotective and neurorestorative in mouse and rat models of PD, supporting the idea that netrin‐1 is implicated in PD pathologies.^[^ ^12^ ^]^ The UNC5C is a canonical transmembrane receptor that contains a death domain (DD) in the C‐terminus.^[^ ^13^ ^]^ After binding to its ligand netrin‐1, the complex plays a crucial role in mediating axon repulsion of neuronal growth cones and cell migration in the developing nervous system.^[^ ^14^ ^]^ UNC5C is also widely expressed in adult CNS neurons. So far, six single nucleotide polymorphisms (SNPs) in UNC5C have been reported to increase the risk of late‐onset AD.^[^ ^15^ ^]^ Recently, a mechanistic study by Hashimoto et al. showed that UNC5C and its T835M mutant in DD trigger neuronal cell death via DAPK1/PKD/ASK1/JNK/NADPH oxidase/caspases pathways.^[^ ^16^ ^]^ In the current work, we show that netrin‐1 is reduced in the brains of aged PD mouse models and PD patients that results in AEP activation, which subsequently cleaves UNC5C receptor at N467 and N547 residues, enhancing neuronal cell death. Blunting AEP cleavage of UNC5C mitigates netrin‐1 deficiency‐elicited dopaminergic neuronal loss, associated with reduced *α*‐Syn pathology. Overexpression of AEP‐truncated UNC5C in the SNpc of *α*‐SNCA mice escalates dopaminergic loss and *α*‐Syn aggregation, augmenting motor dysfunctions.

## 2 Results

### 2.1 Netrin‐1 is Reduced in PD Patient Brains Associated with UNC5C Receptor Cleavage by Active AEP

**Figure 1:**
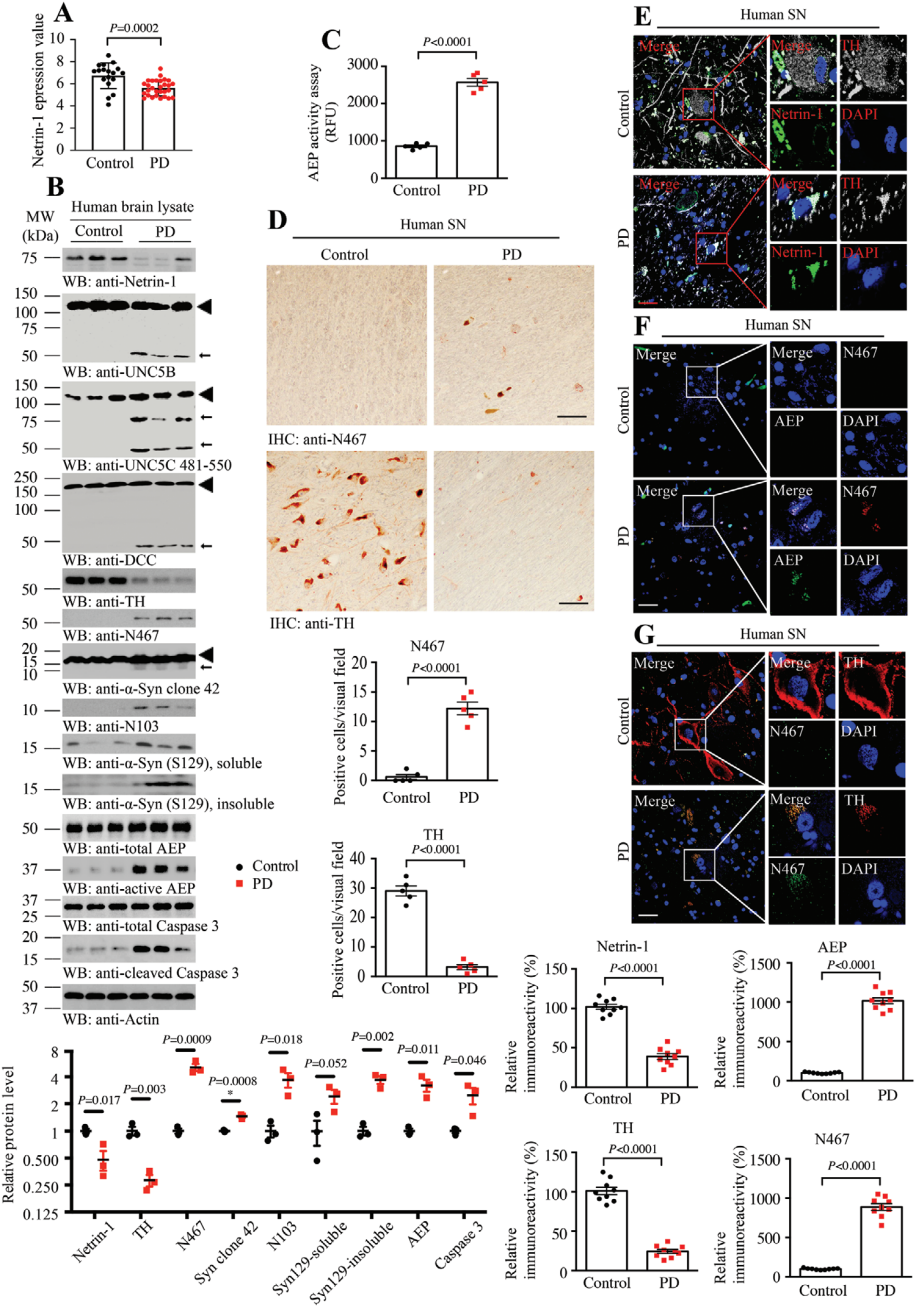
Netrin‐1 reduction in human PD brains, associated with AEP activation and UNC5C cleavage. A) Netrin‐1 expression profiling by array of PD substantia nigra from dataset GDS3129 (reference series: GSE8397). Means + s.e.m. are shown (18 Control, 29 PD cases; unpaired *t*‐test). B) Western blot (WB) showing that netrin‐1 reduction accompanied by AEP and caspase‐3 activation in substantia nigra (SN) samples from subjects with PD. Actin, loading control; MW, molecular weight; ◄, full length; ←, cleaved. The data of WB quantitative analysis are presented as means ± s.e.m.(*n* = 3; 2‐tailed *t*‐test). C) AEP activity assay of the human SN samples from subjects with PD and age‐matched healthy controls. RFU, relative fluorescence units. Means + s.e.m. are shown (*n* = 5; 2‐tailed *t*‐test). D) Immunohistochemistry of UNC5C N467 fragment and TH in SN sections from people with PD and age‐matched healthy controls. Scale bar, 100 µm. Means + s.e.m. are shown (*n* = 5; 2‐tailed *t*‐test). E–G) Immunofluorescence co‐localization analysis was used to detect the expression of netrin‐1, TH, N467, and total AEP. Images are representative of nine sections from three subjects with PD and age‐matched healthy controls. Scale bar, 30 µm. Bar graph shows data as mean ± s.e.m.; 2‐tailed *t*‐test.

Netrin‐1 and its receptors, UNC5C and DCC, are highly expressed in meso‐corticolimbic dopaminergic neurons under physiological conditions.^[^ ^17^ ^]^ We also confirmed their expression in SN, striatum, and primary dopaminergic neurons (Figure S1, Supporting Information). As secreted protein, netrin‐1 is mainly expressed in neurons and astrocytes in the brain (Figure S1C, Supporting Information).^[^ ^18^ ^]^ However, netrin‐1 transcription is substantially reduced in SNs from post‐mortem brain samples of individuals with sporadic PD (control vs PD, 6.74 ± 0.2732 vs 5.63 ± 0.1315, *p* = 0.0002, Expression profiling by array, **Figure** **1**A).^[^ ^19^ ^]^ To explore whether netrin‐1 and its receptors are implicated in PD, we conducted immunoblotting and found that netrin‐1 was robustly attenuated in PD patient brains as compared to age‐matched healthy controls (52% decline, *p* = 0.017, Figure 1B). Moreover, its receptor UNC5B, C, and DCC were prominently fragmented. Noticeably, the proteases including AEP (3.2 times vs control, *p* = 0.011, Figure 1B) and caspase‐3 (2.5 times vs control, *p* = 0.046, Figure 1B) were strongly activated, inversely correlated with conspicuous TH reduction (72% decline, *p* = 0.003, Figure 1B), suggesting extensive dopaminergic neuronal loss. Furthermore, AEP‐truncated *α*‐Syn N103 was increased in PD brains, associated with highly phosphorylated *α*‐synuclein (Figure 1B). Previous studies show that UNC5B is cut by active caspase‐3 at D412, UNC5C at D415, and DCC at D1291 residues, respectively.^[^ ^20^ ^]^ Accordingly, the extra fragmented band around 75 kDa in UNC5C attracted our attention.

To determine the potential cleavage sites in UNC5C receptor, we hypothesized that this proteolytic reaction resulted from AEP protease truncation, because this enzyme is activated in PD and specifically cleaves *α*‐Syn at N103 and promotes its aggregation.^[^ ^6^ ^]^ AEP is an acidosis‐activated asparagine endopeptidase that cleaves substrates under pH 6.0 or lower. To confirm that AEP cleaves UNC5C receptor, we prepared mammalian expressed mGST‐UNC5C proteins. As expected, in vitro cleavage assay with brain lysates from AEP +/+ and −/− mice showed that UNC5C was selectively fragmented under pH 6.0 but not 7.4 in AEP +/+ brains, which was abolished in AEP −/− brains. Introduction of rAEP proteins reconstituted UNC5C fragmentation (Figure S2A, Supporting Information, upper panel). Remarkably, this proteolytic reaction was specifically blocked by AENK but not AEQK tetrapeptide inhibitor for AEP, underscoring that AEP specifically cleaves UNC5C receptor into multiple fragments (Figure S2A, Supporting Information, lower panel). To ascertain that UNC5C is a substrate of AEP, we conducted in vitro cleavage assay, and found that C‐terminal HA‐tagged UNC5C was selectively cleaved by recombinant rAEP proteins at pH 6.0 but not 7.4 (Figure S2B, Supporting Information, top panel). Based on the molecular weight for the fragmented band, we generated several point mutants by changing N into A in UNC5C and found that N467A and N547A strongly reduced UNC5C FL degradation under pH 6.0. It was worth noting that anti‐UNC5C antibody identified the antigen a.a. 481–550 on UNC5C receptor. Hence, neither anti‐HA nor anti‐UNC5C antibody recognized the truncated a.a. 1–467 fragment on immunoblotting. Moreover, these two mutants highly reduced 50 or 75 kDa truncates, respectively, supporting that these two residues might play essential roles in mediating UNC5C fragmentation. Though N519A mutation also diminished these two truncates, it failed to attenuate UNC5C FL decomposition, indicating that this reside might not be a critical cutting site on UNC5C (Figure S2B, Supporting Information). Next, we generated rabbit polyclonal antibodies against N467 and N547 sites and purified the antibodies. Notably, anti‐N467 selectively recognized the truncated UNC5C N467 band (Figure S2C, Supporting Information, middle panel), demonstrating that AEP cleaves UNC5C receptor at N467 residue under pH 6.0. Although anti‐N547 identified the truncated 75 kDa UNC5C fragment, this antibody was not very specific (Figure S2C, Supporting Information, bottom panel). To further verify its specificity, anti‐UNC5C N467 antibody was preincubated with the antigen peptide (UNC5C 459–467) or nonspecific peptide before immunohistochemistry (IHC). The signal was blocked by UNC5C 459–467 peptide but not the nonspecific one (Figure S2D, Supporting Information). Remarkably, AEP selectively cleaved UNC5C but not other UNC5s or DCC receptors (Figure S2E–H, Supporting Information), supporting the receptor cleavage specificity by AEP.

Interestingly, the purified polyclonal anti‐N467 antibody selectively recognized truncated human UNC5C fragment in human PD patient SN lysates (Figure 1B). As expected, AEP was substantially activated in PD brains as compared to healthy controls (3 times vs control, Figure 1C). Tyrosine hydroxylase (TH) is the rate‐limiting enzyme in the synthesis of dopamine, and usually regarded as a molecular marker of dopaminergic neurons. IHC staining revealed that UNC5C N467 fragment was evidently increased in PD brains versus control brains, associated with great reduction of TH staining (Figure 1D), indicating that dopaminergic neuronal loss in PD brains is accompanied with robust AEP activation and UNC5C proteolytic fragmentation. To further test this notion, we performed immunofluorescent (IF) staining on human brain sections. Compared with control, netrin‐1 was decreased in PD brains, associated with robust TH reduction (Figure 1E). In alignment with IB observations, UNC5C N467 immunoreactivity was strongly elevated in PD brains, coupled with demonstrable AEP escalation and dopaminergic neuronal loss (Figure 1F,G). Quantification is shown in the lower panels of Figure 1G. Therefore, these studies support that netrin‐1 is deficient in PD patient brains and induces AEP activation and UNC5C proteolytic fragmentation.

To assess whether these events could also occur in the PD neurotoxin‐induced biological effects, we chronically treated human *α*‐SNCA transgenic mice with rotenone via oral administration. Quantitative RT‐PCR showed that rotenone significantly augmented mRNAs of these netrin‐1 receptors. In contrast, netrin‐1 expression was greatly repressed (Figure S3A, Supporting Information). Immunoblotting revealed that rotenone sturdily suppressed netrin‐1 protein levels, conversely coupled with elevation of its receptors, which were strongly truncated. These effects were accompanied by evident TH loss and AEP activation, leading to robust *α*‐Syn N103 fragmentation. In alignment with previous report, *α*‐Syn FL was clearly escalated upon rotenone treatment (Figure S3B, Supporting Information). Titration assay showed that rotenone dose‐dependently triggered netrin‐1 reduction and netrin receptors truncation in dopaminergic SH‐SY5Y cells. Consequently, both AEP and caspase‐3 were activated in a concentration‐dependent manner (Figure S3C, Supporting Information). Hence, netrin‐1 reduction in PD patient brains or rotenone‐treated mice and cells is associated with AEP activation and netrin‐1 receptors proteolytic fragmentation.

To assess whether netrin‐1 reduction is responsible for these biological effects, we prepared primary neuronal cultures and neutralized medium secreted netrin‐1 using its monoclonal antibody 2F5 or recombinant proteins DCC‐4Fbn. Netrin‐1 withdrawal provoked evident AEP proteolytic activation, which was validated with its substrate APP cleavage, leading to APP N585 truncation and resultant APP C586 escalation. As expected, caspase‐3 was activated upon netrin withdrawal. Notably, UNC5B, C, and DCC all were strongly truncated with a similar pattern (Figure S4A, Supporting Information). Previous studies show that all of these netrin‐1 receptors are caspase‐3 substrates, and UNC5B is cut by active caspase‐3 at D412, UNC5C at D415, and DCC at D1291, respectively.^[^ ^20^ ^]^ To clarify the biological relationship between AEP and caspase‐3 in cleaving these receptors, we employed AEP −/− neurons. As compared to wild‐type neurons, netrin‐1 withdrawal elicited receptors’ fragmentation was strongly reduced. Remarkably, caspase‐3 activation was also decreased, indicating that AEP somehow regulates caspase‐3 proteolytic activation (Figure S4B, Supporting Information). It is worth noting the 75 kDa truncated UNC5C band completely disappeared in AEP‐null neurons, whereas all other netrin receptors’ fragments were detectable, though their abundance was greatly diminished in AEP KO neurons. These findings indicate that the 75 kDa band in UNC5C might be solely cleaved by active AEP, whereas other netrin‐1 receptors’ fragments mainly resulted from active caspase‐3 cleavage (Figure S4B, Supporting Information). To ascertain that AEP mediates UNC5C cleavage in primary neurons, we infected primary neuronal cultures with viral vectors expressing enzymatic‐dead C189S mutant. Compared to control viral vector, netrin‐1 neutralization‐incurred AEP activation was completely blocked by C189S mutant. As a consequence, caspase‐3 activation was attenuated. The positive control AEP‐truncated APP N585 and C586 signals were totally blocked in AEP C189S‐infected neurons. C189S mutation in AEP leads to the inactivation of AEP while keeping the binding ability with its substrates. Accordingly, both UNC5B and C proteolytic cleavage were highly decreased. Again, the 75 kDa band was selectively eradicated, supporting that this truncate in UNC5C is specifically cleaved by active AEP (Figure S4C, Supporting Information).

### 2.2 Netrin‐1 is Age‐Dependently Reduced in *α*‐SNCA Transgenic Mice, Enhancing AEP Activation and UNC5C Cleavage

**Figure 2:**
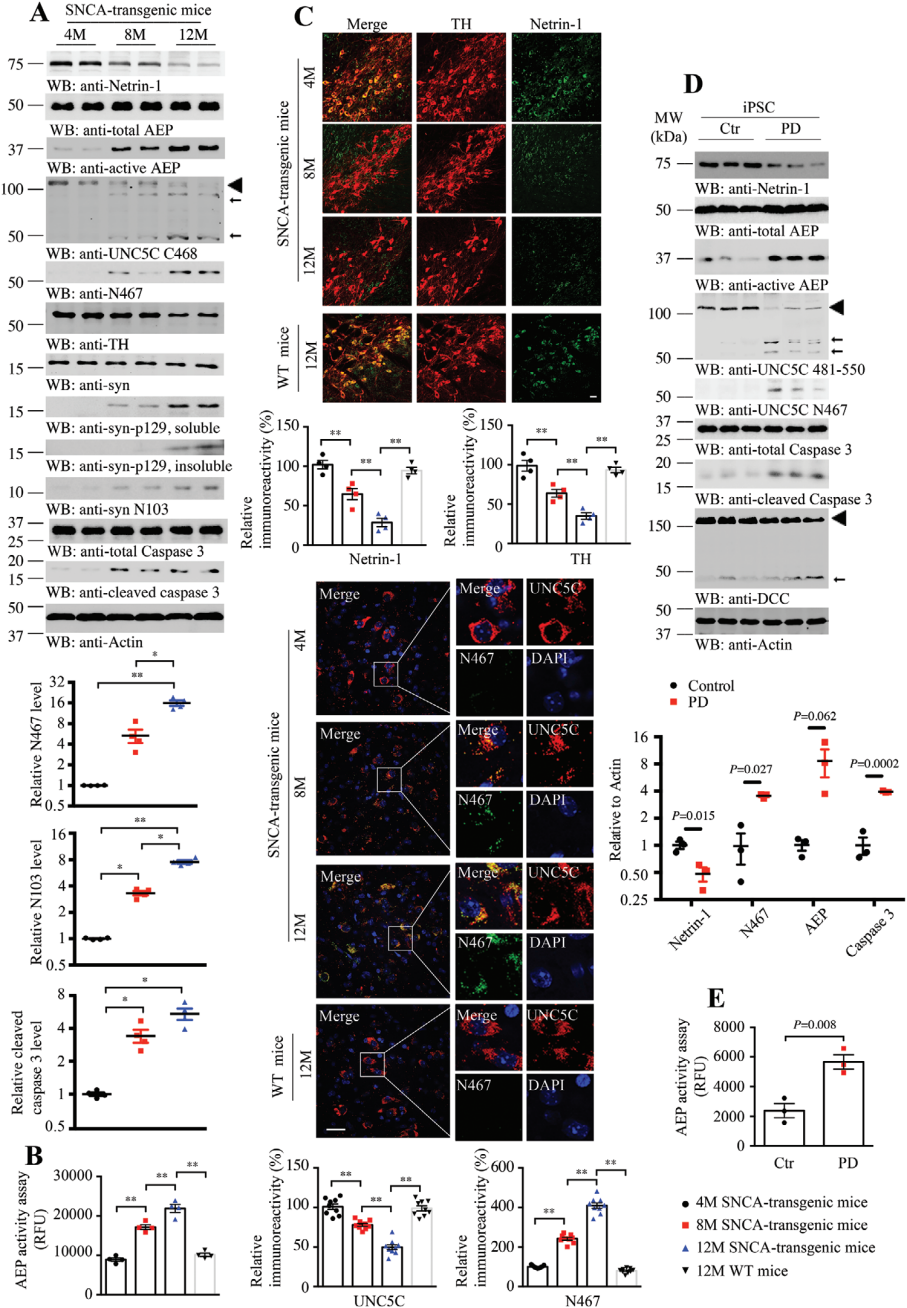
Netrin‐1 is reduced and active AEP cleaves UNC5C in SNCA‐transgenic mice and induced pluripotent stem cells. A) Western blot (WB) showing the processing of netrin‐1 deprivation and UNC5C cleaved by AEP in an age‐dependent manner in human SNCA‐transgenic mice. Actin, loading control; MW, molecular weight; ◄, full length; ←, cleaved. The data of WB quantitative analysis are presented as means ± s.e.m.; *n* = 4; *p < 0.05, **p < 0.01 by one‐way ANOVA followed by Tukey's multiple‐comparisons test. B) AEP activity in the SN of SNCA‐transgenic mice at different ages. Data are mean ± s.e.m.; *n* = 4 mice per group; **p < 0.01 by one‐way ANOVA followed by Tukey's multiple‐comparisons test; RFU, relative fluorescence units. C) Immunofluorescence co‐localization analysis was conducted with various antibodies against netrin‐1 and TH, UNC5C 812–823 and N467 in SNCA‐transgenic, and WT mice at different ages. Images are representative of nine sections from three subjects. Scale bar, 30 µm. D) Western blot (WB) showing the processing of netrin‐1 deprivation and UNC5C cleaved by AEP in iPSC‐differentiated neurons. Actin, loading control; ◄, full length; ←, cleaved. The data of WB quantitative analysis are presented as means ± s.e.m. (*n* = 3, 2‐tailed *t*‐test). E) AEP activity of iPSC. Data are mean ± s.e.m. (*n* = 3, 2‐tailed *t*‐test); AFU, arbitrary fluorescence units.

Although *α*‐SNCA transgenic mice do not recapitulate all the features of human disease, they temporally develop *α*‐synuclein pathology and motor dysfunctions. Accordingly, we monitored netrin‐1 expression in human *α*‐SNCA transgenic mice and found that its levels were repressed in an age‐dependent manner. By contrast, AEP was progressively activated, tightly correlated with gradual fragmentation of UNC5C, and coupled with the escalation of its UNC5C N467 truncate (8 month vs 12 month, 4.67 times vs 16.56 times, *p* < 0.05, **Figure** **2**A). Consistently, both *α*‐Syn N103 and p‐*α*‐Syn S129 were increased, fitting with enhanced active AEP concentrations, while TH loss was progressively elevated (18% decline at 8‐month and 45% decline at 12‐month compared to the levels at 4‐month). Again, caspase‐3 was also temporally activated (3.42 times at 8‐month and 5.43 times at 12‐month compared to the levels at 4‐month, Figure 2A). AEP enzymatic activities were steadily augmented as the age increased in *α*‐SNCA mouse brains (Figure 2B). IF co‐staining demonstrated that both netrin‐1 and TH staining intensities were gradually declined, in agreement with IB observations (Figure 2C upper). The co‐staining of UNC5C 812–823 and N467 showed the colocalization of them in the SN region and the fragmentation of UNC5C increased with aging (Figure 2C middle). To further investigate these biological effects, we employed human induced pluripotent stem cell (iPSC)‐derived) neurons from PD patients and control subjects. IB analysis revealed that netrin‐1 was greatly decreased in PD human neurons versus those derived from healthy controls (52% decline, *p* = 0.015, Figure 2D), associated with robust AEP (3.5 times, *p* = 0.003, Figure 2D) and caspase‐3 (3.9 times, *p* = 0.0002, Figure 2D) activation, which coupled with UNC5C proteolytic cleavage (Figure 2D). Quantification with AEP enzymatic assay supported that AEP was significantly activated in PD neurons versus controls (Figure 2E). Therefore, netrin‐1 is reduced in human *α*‐SNCA transgenic PD mice in an age‐dependent manner, eliciting AEP activation and UNC5C N467 fragmentation.

### 2.3 AEP Cleavage of UNC5C Escalates its Neuronal Cell Death Activities

**Figure 3:**
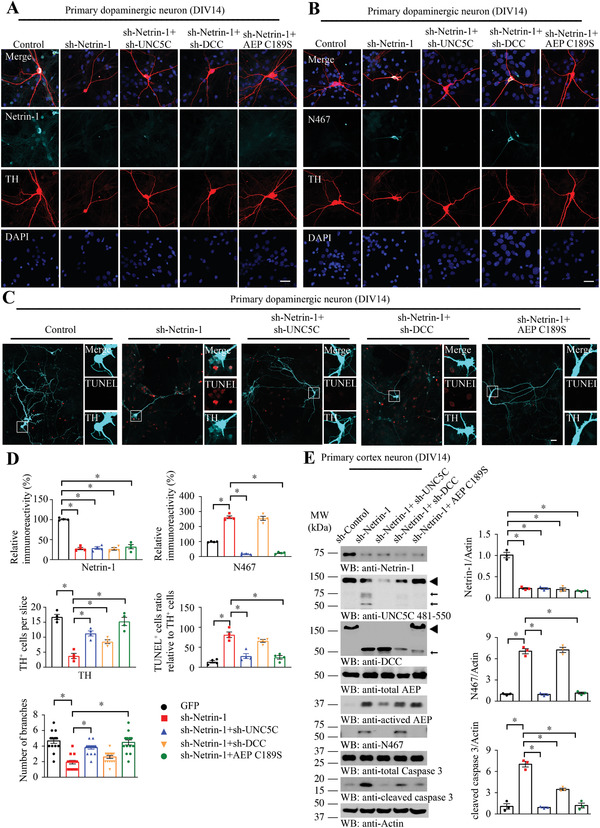
Netrin‐1 depletion in primary dopaminergic neurons induces UNC5C fragmentation and neuronal apoptosis. Primary dopaminergic neurons and cortex neurons at days in vitro (DIV) 7 were infected with AAV viral vectors expressing sh‐Netrin‐1, sh‐UNC5C, sh‐DCC, AEP C189S, or combined of them. 7 days after transfection, neurons were stained with different antibodies to show the effects of netrin‐1 depletion. A) Immunofluorescent signals of TH (red) and netrin‐1 (cyan). The nuclei were stained with DAPI. B) Immunofluorescent signals of TH (red) and UNC5C N467 (cyan). The nuclei were stained with DAPI. C) TUNEL assay was conducted to detect the apoptotic effect of different treatments in primary dopaminergic neurons marked with TH (cyan). Scale bar, 30 µm. D) Quantification of the immunofluorescent signals in (A–C). Data are mean ± s.e.m., representatives of 3 independent experiments (15 neurons for branches analysis); *p < 0.05 by one‐way ANOVA followed by Tukey's multiple‐comparisons test. E) Primary cortical neurons at DIV 14 were collected for detecting netrin‐1, AEP, UNC5C, DCC, and caspase 3 expression by WB. ◄, full length; ←, cleaved. The data of WB quantitative analysis are presented as means ± s.e.m.; *n* = 3; *p < 0.05 by one‐way ANOVA followed by Tukey's multiple‐comparisons test.

Previous study shows that netrin receptors act as dependence receptors, namely, they trigger apoptosis in the absence of trophic ligand, whereas they promote cell survival when netrins are available and bind to the receptors.^[^ ^21^ ^]^ To show the direct effects of netrin‐1 and its receptors in dopamine neurons, we infected primary dopaminergic neurons at 7 days in vitro (DIV) with AAV viral vectors expressing sh‐Netrin‐1, sh‐UNC5C, sh‐DCC, AEP C189S, or combinations of them. 7 days after infection, netrin‐1 expression was significantly reduced, accompanied with prominent UNC5C cleavage by AEP (**Figure** **3**A,B). We further conducted TUNEL assay to detect the neuronal apoptosis and found that netrin‐1 deletion induced dopaminergic neuronal apoptosis, which could be blunted by knockdown of UNC5C or blockade of AEP activity using inactive C189S mutant (Figure 3C,D). Immunoblotting validated these observations in primary cortical cultures infected with these constructs (Figure 3E).

**Figure 4:**
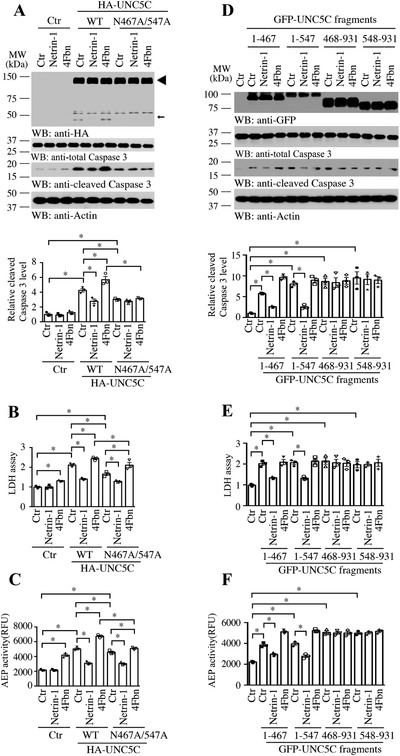
Fragments of UNC5C cleaved by AEP enhance SH‐SY5Y cell death. SH‐SY5Y cells were transfected with UNC5C WT, N467/547A mutant, or truncated plasmids. 24 h after transfection, cells were treated with 0.5 µg mL^−1^ netrin‐1, 10 µg mL^−1^ (DCC‐4Fbn, to block netrin‐1), or control vehicle (Ctr) for another 24 h. A,D) Cell lysates were probed with anti‐HA or anti‐GFP tag antibody to detect the expression of above plasmids by Western blot (WB). Cleaved caspase 3 was used to reveal their toxicity effects. Actin, loading control; MW, molecular weight. The data of WB quantitative analysis are presented as means ± s.e.m.; *n* = 3; *p < 0.05 by 2‐way ANOVA and Bonferroni's post hoc test. B,E) Mediums were collected for LDH assay. C,F) AEP activity in the cell lysates. RFU, relative fluorescence units. For (C–F), data are mean ± s.e.m., representatives of 3 independent experiments; *p < 0.05 by 2‐way ANOVA and Bonferroni's post hoc test.

To assess the biological role of AEP cleavage of UNC5C in triggering cell death, we transfected dopaminergic SH‐SY5Y cells with HA‐tagged UNC5C WT or AEP‐resistant N467/547A double mutant, followed by trophic netrin‐1 treatment or netrin deprivation using DCC‐4Fbn recombinant proteins. Immunoblotting showed that overexpression of UNC5C WT strongly provoked caspase‐3 activation (4.3 times vs control, *p* < 0.05, **Figure** **4**A), associated with UNC5C cleavage, which was significantly decreased by netrin‐1 treatment (35% decline, *p* < 0.05, Figure 4A) and enhanced by netrin withdrawal (1.3 times, *p* < 0.05, Figure 4A), respectively. Strikingly, the truncated UNC5C band was completely eliminated from AEP‐resistant mutant transfected cells, indicating the fragmented band resulted from AEP cleavage. Moreover, active caspase‐3 was also prominently attenuated in these cells (Figure 4A). LDH assay showed that DCC‐4Fbn elicited significant cell death in control cells, and its cell death stimulatory effect was further enhanced in the presence of UNC5C WT, whereas it was significantly mitigated when cells were transfected with AEP‐uncleavable UNC5C mutant (Figure 4B). As expected, netrin‐1 substantially repressed UNC5C‐triggered cell death regardless of WT or double mutant (Figure 4B). Quantification showed that AEP enzymatic activities exhibited a similar format to LDH activities, suggesting that AEP activation contributes to cell death effects (Figure 4C).

To address whether AEP cleavage of UNC5C affects its effect in cell death, we transfected SH‐SY5Y cells with AEP‐truncated N‐terminal or C‐terminal fragments, respectively, followed with trophic netrin‐1 treatment or netrin deprivation. Again, all of these fragments significantly escalated caspase‐3 activation in transfected cells compared with control vector, which was repressed by netrin‐1 treatment, and DCC‐4Fbn treatment further augmented caspase‐3 activation (Figure 4C). LDH assay indicated cell death activities echoed with caspase‐3 activation. Notably, the cells transfected with UNC5C extracellular fragments (1–467; 1–547) were responsive to netrin‐1 treatment and inhibited cell death. By contrast, its intracellular fragments (468–931, 548–931) strongly stimulated cell death, refractory to any treatment, no matter the transfected cells were treated with netrin‐1 or DCC‐4Fbn (Figure 4D,E). AEP enzymatic activities oscillated with LDH activities (Figure 4F). Thus, our data support that blockade of AEP cleavage of UNC5C decreases cell death, and AEP‐truncated intracellular UNC5C fragment strongly stimulates cell death.

### 2.4 Deletion of Netrin‐1‐Induced Dopaminergic Neuronal Loss is Mediated by AEP Cleavage of UNC5C

**Figure 5:**
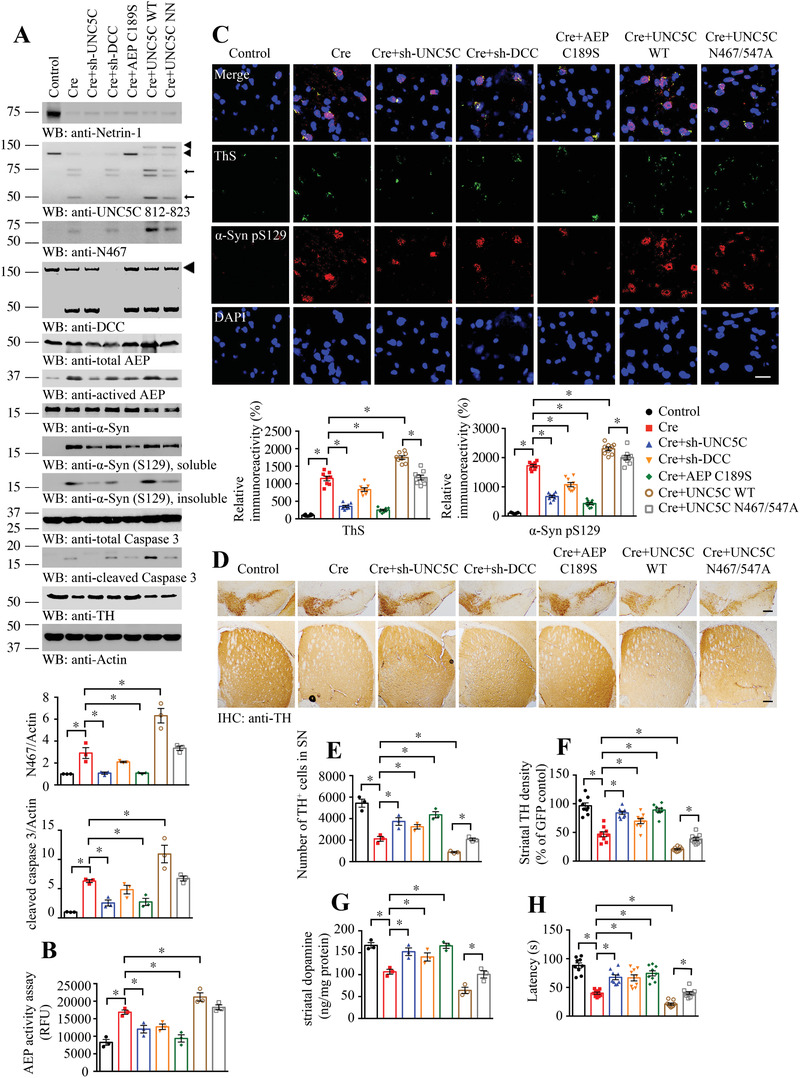
Netrin‐1 deletion in Netrin f/f mice induces dopamine neuronal loss and motor deficits via AEP‐cleaved UNC5C receptor. A) Western blot (WB) showing the processing of netrin‐1 deprivation by injecting AAV‐Cre viral vectors into SN and UNC5C fragmentation induced by subsequent AEP activation in Netrin f/f mice. And further verify the role of netrin‐1 receptors and AEP coupled with regulating UNC5C, DCC expression or inactivating AEP. SN lysates were detected with various indicated antibodies. Actin, loading control. The viral vectors were injected into both sides of SN. The upper◄, GFP‐UNC5C; the lower◄, endogenous full length UNC5C; ←, cleaved UNC5C. The data of WB quantitative analysis are presented as mean ± s.e.m.; *n* = 3 mice. *p < 0.05 by one‐way ANOVA followed by Tukey's multiple‐comparisons test. B) AEP activity in the SN of Netrin f/f mice. Data are mean ± s.e.m.; *n* = 3 mice. RFU, relative fluorescence units. C) Immunofluorescent signals of Thioflavin S (ThS, green) and anti‐*α*‐synuclein pS129 (*α*‐syn pS129, red) were detected. And the nuclei were stained with DAPI. Scale bar, 20 µm. D) Immunohistochemistry staining was performed to analyze TH expression in the SN and striatum of above animals. Scale bar, 200 µm. E,F) Quantification of TH+ neuron numbers in SN and TH signal density in the striatum. Data are mean ± s.e.m.; *n* = 3 mice. G) Concentrations of striatal dopamine were determined by HPLC. Data are mean ± s.e.m.; *n* = 3 mice. H) Rotarod was conducted to detect the motor function of above mice by a blinded observer 3 months after the viral vector injection. Data are mean ± s.e.m.; *n* = 9 mice per group. *p < 0.05 by one‐way ANOVA followed by Tukey's multiple‐comparisons test.

Netrin‐1 is greatly diminished in the brains of PD patients, and it is also evidently reduced in both neurotoxin rotenone‐treated mice, cells, and *α*‐SNCA transgenic mice. To investigate whether netrin‐1 reduction elicits dopaminergic neuronal loss is implicated in PD pathogenesis, we employed netrin flox/flox mice and injected control or AAV‐Cre viral vector into the SN region of the mice. In Cre viral vector injected brains, we co‐injected with viral vector expressing shRNAs against UNC5C or DCC, separately. Moreover, we also co‐administrated viral vectors expressing enzymatic‐dead AEP C189S, UNC5C WT, or AEP‐resistant double mutant, respectively. 3 months after viral injection, we monitored the constructs expression and biological effects by immunoblotting with the brain lysates from the SN regions. Cre viral vector successfully decreased endogenous netrin‐1 in the brains and triggered detectable AEP activation and UNC5C cleavage at N467. Fitting with these findings, both soluble and insoluble p‐*α*‐Syn S129 was evidently elevated, which was inversely correlated with TH reduction. Consequently, caspase‐3 was also significantly activated and DCC was truncated, when netrin‐1 was deleted. Depletion of UNC5C with its shRNA abrogated N467 cleavage and diminished AEP and caspase‐3 activation, associated with TH increase and p‐S129 reduction (**Figure** **5**A). We made a similar observation with shRNA against DCC. As expected, C189S mutant blocked netrin‐1 deletion‐elicited AEP activation and UNC5C N467 fragmentation. Consequently, caspase‐3 activation was notably reduced and TH level was elevated, and p‐S129 signal was subdued. However, overexpression of UNC5C WT in netrin‐1‐deleted brains strongly escalated UNC5C cleavage and N467 fragmentation, associated with prominent active AEP and caspase‐3 (Cre + UNC5C WT vs Cre, 10.9 vs 4.6, *p* < 0.05, Figure 5A), and DCC was evidently truncated. Consistent with these events, TH was robustly reduced, whereas p‐S129 activities were increased. Noticeably, all of these effects were ameliorated in the presence of uncleavable UNC5C double mutant compared to WT UNC5C (Figure 5A). Quantification revealed that AEP enzymatic activities tightly coupled with active AEP abundances in IB analysis (Figure 5B). To assess PD‐related pathologies in netrin‐1‐deleted SN regions, we conducted IF co‐staining with anti‐p‐*α*‐Syn S129 and Thioflavin S (ThS). Compared with control viral vector, netrin‐1 deletion elicited robust p‐*α*‐Syn S129 signals, which were co‐localized with ThS, suggesting that aggregated *α*‐Syn forms Lewy body‐like structures. Interestingly, these activities were mitigated when UNC5C or DCC receptor was deleted. The maximal reduction occurred, when C189S inactive AEP mutant was overexpressed. By contrast, overexpression of UNC5C WT strongly enhanced netrin‐1 deficiency‐elicited p‐*α*‐Syn S129, which was attenuated by AEP‐uncleavable mutant (Figure 5C). The p‐*α*‐Syn S129 fluorescent signals tightly correlated with IB findings.

Next, we performed IHC staining with the SN and striatal sections from netrin‐1 deleted mice, and found that TH activities conversely coupled with p‐*α*‐Syn S129 signals in both regions. Quantification of TH‐positive dopaminergic neurons with stereological counting tightly correlated with TH IHC signals. Netrin‐1 deletion significantly decreased dopaminergic neurons as compared to control, which were alleviated when DCC or UNC5C was knocked down, indicating that netrin deficiency elicited dopaminergic neuronal loss is mediated by the netrin receptors. Moreover, TH degeneration was repressed when AEP was blocked by inactive C189S mutant. As expected, overexpression of UNC5C WT exacerbated netrin‐1‐elicited dopaminergic neuronal loss, which was alleviated by blocking AEP‐mediated UNC5C proteolytic cleavage (Figure 5D–F). IF co‐staining with brain sections validated that TH immuno‐activities inversely coupled with AEP‐truncated UNC5C N467 fluorescent intensities (Figure S5A, Supporting Information), in alignment with the observations in Western blotting. We also used another dopaminergic neuronal marker, VMAT2, to further verify the neuronal loss, and found that the results were correlated with TH staining (Figure S5B, Supporting Information). IHC staining with anti‐p‐*α*‐Syn‐S129 revealed that netrin‐1 deletion incurred robust p‐*α*‐Syn S129 activities, which were mitigated when UNC5C or DCC was knocked down. The phosphorylation signals were greatly repressed when inactive C189S was expressed. However, p‐*α*‐Syn S129 was further escalated when UNC5C WT was co‐expressed, which was alleviated in the presence of AEP‐uncleavable UNC5C mutant (Figure S5C, Supporting Information). IF co‐staining with 5G4 antibody, which specifically recognizes aggregated *α*‐Syn inclusion, and ThS, a fluorescent dye visualizing misfolded fibrillary aggregates, tightly co‐localized in the remnant dopaminergic neurons and exhibited similar patterns as p‐*α*‐Syn S129 (Figure S5D, Supporting Information). HPLC analysis with striatal tissues showed that dopamine concentrations were consistent with TH activities (Figure 5G). Rotarod behavioral test revealed that the locomotor activities mirrored the TH signals (Figure 5H). Together, these data suggest that netrin‐1 deletion in the SN triggers AEP activation, leading to UNC5C fragmentation and dopaminergic cell death.

### 2.5 Knockdown of Netrin‐1 Triggers Dopaminergic Neuronal Loss in *α*‐SNCA Mice via Both UNC5C and DCC Receptors

**Figure 6:**
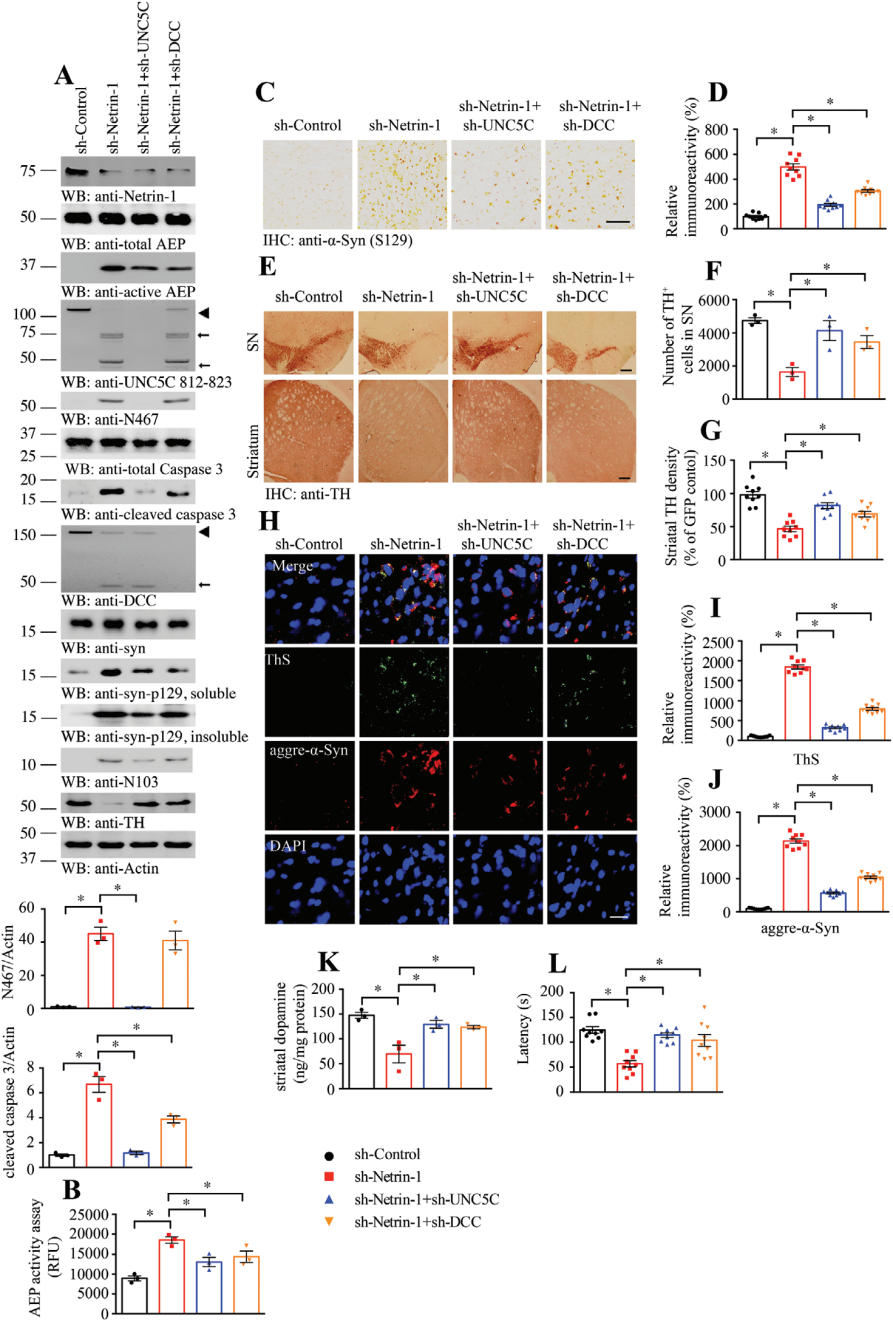
Netrin‐1 depletion in SNCA transgenic mice induces neuronal loss and motor deficits, ameliorated by UNC5C knockdown. A) Western blot (WB) showed that netrin‐1 depletion by injecting AAV‐Netrin‐1 viral vector into SN induced UNC5C fragmentation by AEP activation in *α*‐SNCA transgenic mice. SN lysates were detected with various indicated antibodies. Actin, loading control. The viral vectors were injected into both sides of SN. ◄, full length; ←, cleaved. The data of WB quantitative analysis are presented as mean ± s.e.m.; *n* = 3 mice. *p < 0.05 by one‐way ANOVA followed by Tukey's multiple‐comparisons test. B) AEP activity in the SN of *α*‐SNCA transgenic mice. Data are mean ± s.e.m.; *n* = 3 mice per group. RFU, relative fluorescence units. C,D) Alpha‐synuclein pS129 (*α*‐syn pS129) immunostaining in the SN of above mice. Scale bar, 500 µm. E) Immunohistochemistry staining was performed to analyze TH expression in the SN and striatum of above animals. Scale bar, 200 µm. F,G) Quantification of TH+ neuron numbers in SN and TH signal density in the striatum. Data are mean ± s.e.m.; *n* = 3 mice per group. H–J) Immunofluorescent signals of Thioflavin S (ThS, green) and anti‐aggregated *α*‐synuclein (aggre‐*α*‐syn, red) in SN were detected. And the nuclei were stained with DAPI. Scale bar, 20 µm. Data are mean ± s.e.m.; *n* = 3 mice per group. K) Concentrations of striatal dopamine were determined by HPLC. Data are mean ± s.e.m.; *n* = 3 mice per group. L) Rotarod was conducted to detect the motor function of above mice by a blinded observer 3 months after the viral vectors injection. Data are mean ± s.e.m.; *n* = 9 mice per group. *p < 0.05 by one‐way ANOVA followed by Tukey's multiple‐comparisons test.

To further investigate the role of netrin‐1 deficiency in triggering PD pathologies, we deleted netrin‐1 in the SN regions of human *α*‐SNCA transgenic mice (3 months old) by injecting AAV viral vector expressing its specific shRNA, and this pooled siRNA could significantly improve interference efficiency to ensure specific netrin eradication. Some of the mice were co‐injected with shRNAs against UNC5C or DCC, respectively, or Scramble AAV siRNA virus as control. 3 months later, we examined viral expression efficiency and the biological effects by immunoblotting with SN lysates. Compared with controls, netrin‐1 AAV‐shRNA strongly eradicated endogenous netrin‐1, which was accompanied with robust AEP (2.1 times vs control, *p* < 0.05, **Figure** **6**A) and caspase‐3 (6.7 times vs control, *p* < 0.05, Figure 6A) activation. As a consequence, *α*‐Syn N103 and UNC5C N467 fragmentation were evidently escalated. Subsequently, p‐*α*‐Syn S129 was highly elevated, which was inversely coupled with dramatic reduction of TH activities, indicating extensive dopaminergic neuronal loss, which was associated with demonstrable DCC apoptotic truncation. As expected, knocking down either of UNC5C or DCC with the specific shRNA clearly deleted the corresponding receptor and alleviated the biological effects, leading to partial TH restoration. In alignment with these findings, active AEP and caspase‐3 were also decreased as compared with netrin‐1 knockdown alone, indicating that these receptors somehow mediate the netrin deficiency‐elicited biological effects (Figure 6A). AEP enzymatic assay showed that depletion of DCC or UNC5C significantly reduced AEP activation triggered by netrin‐1 deprivation (Figure 6B). IHC staining revealed that netrin‐1 deletion induced robust p‐*α*‐Syn S129 in the SN brain sections, and knockdown of UNC5C appeared to decrease p‐S129 signals more efficiently as compared to DCC deletion (Figure 6C,D). TH staining with both the SN and striatal sections exhibited a similar effect, and netrin‐1 deletion induced substantial dopaminergic neuronal loss, which was rescued more effectively with UNC5C depletion than DCC depletion (Figure 6E–G), indicating that netrin‐1 deprivation‐elicited dopaminergic neuronal loss is presumably mediated more via UNC5C than DCC receptor. In addition, IF co‐staining with antibodies against TH and UNC5C N467 revealed that TH was strongly reduced in the SN region, when netrin‐1 was deleted as compared to the control, which was inversely correlated with abundant UNC5C N467 cleavage. Thus, AEP is strongly activated and cleaves UNC5C at N467, promoting dopaminergic neuronal cell death. These effects were mitigated when netrin receptor DCC or UNC5C was knocked down. UNC5C depletion substantially diminished N467 cleavage by AEP, leading to alleviation of dopaminergic neuronal loss (Figure S6A, Supporting Information). VMAT2 staining also confirmed the similar results with TH staining (Figure S6B, Supporting Information).

To investigate PD pathologies in *α*‐SNCA mice after netrin‐1 deletion, we performed IF co‐staining with ThS and 5G4 antibody, which specifically recognizes aggregated *α*‐Syn. Extensive aggregated *α*‐Syn were co‐stained with ThS in netrin‐1‐deleted SN regions, indicating that netrin‐1 deficiency elicits the Lewy body‐like inclusions in *α*‐SNCA transgenic mice. These events were significantly reduced when netrin receptors were knocked down with UNC5C deletion exhibiting more prominent effect than DCC deletion (Figure 6H–J). Fitting with aggregated *α*‐Syn IF staining observations, p‐*α*‐Syn S129/ThS co‐staining also supported that netrin‐1 deletion‐incurred p‐*α*‐Syn S129 activities were significantly ameliorated when netrin receptor DCC or UNC5C was knocked down (Figure S6C, Supporting Information). Quantitative analysis showed that netrin‐1 deprivation significantly reduced dopamine levels in *α*‐SNCA transgenic mice, which were restored when UNC5C or DCC receptor was deleted (Figure 6K). Consistent with dopaminergic neuronal recovery and DA restoration, results from the Rotarod behavioral test demonstrated that deletion of netrin‐1 receptor significantly alleviated netrin‐1 deprivation‐triggered motor deficits in *α*‐SNCA mice (Figure 6L). Hence, knockdown of netrin‐1 triggers dopaminergic neuronal loss in *α*‐SNCA mice via its receptors UNC5C and DCC.

### 2.6 Overexpression of AEP‐Truncated UNC5C Intracellular Domain in *α*‐SNCA Transgenic Mice Augments Dopaminergic Neuronal Loss and Motor Deficits

**Figure 7:**
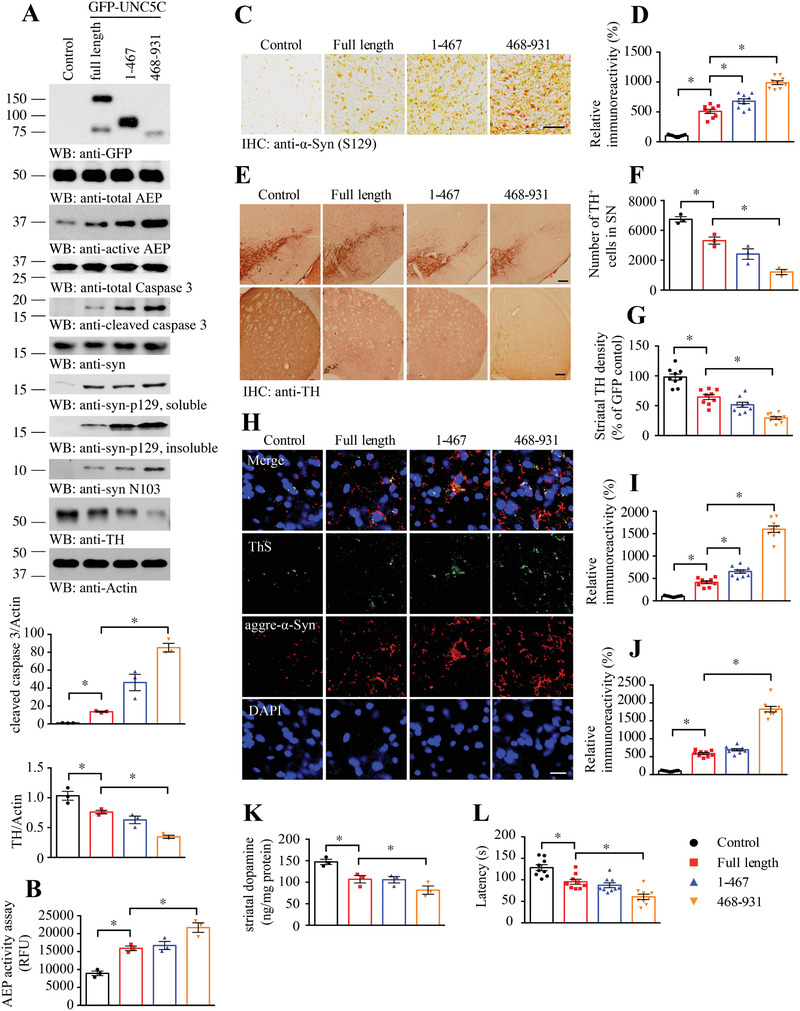
UNC5C fragments induce dopamine neuronal loss and motor deficits in SNCA transgenic mice. A) Western blot (WB) showing the expression of UNC5C full length, the UNC5C 1–467 and 468–931 fragments in SN by injecting correlative LV vectors into SN of *α*‐SNCA transgenic mice. SN lysates were detected with various indicated antibodies. Actin, loading control. The viral vectors were injected into both sides of SN. Control: LV‐GFP. The data of WB quantitative analysis are presented as mean ± s.e.m.; *n* = 3 mice. *p < 0.05 by one‐way ANOVA followed by Tukey's multiple‐comparisons test. B) AEP activity in the SN of SNCA‐transgenic mice. Data are mean ± s.e.m.; *n* = 3 mice per group. RFU, relative fluorescence units. C,D) Alpha‐synuclein pS129 (*α*‐syn pS129) immunostaining in the SN of above mice. Scale bar, 500 µm. E–G) Immunohistochemistry staining was performed to analyze TH expression in the SN and striatum of above animals. Scale bar, 200 µm. Quantification of TH+ neuron numbers in SN and TH signal density in striatum. Data are mean ± s.e.m.; *n* = 3 mice per group. H–J) Immunofluorescent signals of Thioflavin S (ThS, green) and anti‐aggregated *α*‐synuclein (aggre‐*α*‐syn, red) were detected. And the nuclei were stained with DAPI. Scale bar, 20 µm. Data are mean ± s.e.m.; *n* = 3 mice per group. K) Concentrations of striatal dopamine were determined by HPLC. Data are mean ± s.e.m.; *n* = 3 mice per group. L) Rotarod was conducted to detect the motor function of above mice by a blinded observer 3 months after the viral vectors injection. Data are mean ± s.e.m.; *n* = 9 mice per group. *p < 0.05 by one‐way ANOVA followed by Tukey's multiple‐comparisons test.

To define the pathological roles of AEP‐truncated UNC5C in PD pathogenesis, we injected the SN regions of *α*‐SNCA mice (3 months old) with AAV viral vector expressing GFP‐tagged UNC5C FL or N‐terminal or C‐terminal fragment after AEP cleavage. 3 months later, we examined the viral expression efficiency and biological consequences via immunoblotting using the SN lysates. As expected, the administrated constructs were robustly expressed, which significantly provoked intensive AEP and caspase‐3 activation (**Figure** **7**A). Consequently, TH was strongly decreased in FL UNC5C expressed brain as compared with control, which was further reduced when AEP‐truncated UNC5C was expressed with C‐terminus more prominent than N‐terminus. These effects were consistent with in vitro cell death stimulatory effects (Figure 4D–F). As a result, human *α*‐Syn N103 was potently escalated, in agreement with active AEP activities, correlating with evident p‐*α*‐Syn S129 signals (Figure 7A). AEP enzymatic activities closely fit with active AEP fragment in the IB analysis (Figure 7B). IHC staining with p‐S129 antibody showed that overexpression of UNC5C FL in the SN clearly elevated p‐S129 activities, which were further enhanced by AEP‐truncated fragments with the C‐terminus more prominent than the N‐terminus (Figure 7C,D), in alignment with Western blotting results. IHC staining with anti‐TH demonstrated that TH signals inversely coupled with p‐S129 activities in both the SN and striatal sections (Figure 7E–G), supporting that dopaminergic loss is the most by AEP‐truncated UNC5C 468–931. IF co‐staining indicated that p‐*α*‐Syn S129 was elevated, correlating with escalated ThS signals in the SN sections (Figure S7A, Supporting Information). VMAT2 staining revealed dopaminergic neuronal loss in the same region (Figure S7B, Supporting Information). Consistent with these findings, Lewy body‐like inclusions in the SN as viewed with 5G4/ThS co‐staining positively correlated with dopaminergic neuronal loss (Figure 7H–J). Quantification revealed that dopamine concentrations in the striatum tightly fit TH+‐ dopaminergic neurons (Figure 7K). Motor behavioral test showed that UNC5C overexpression significantly decreased the motor activities, which was further reduced when AEP‐truncated UNC5C terminus was expressed in the SN of human *α*‐SNCA transgenic mice (Figure 7L). Therefore, overexpression of AEP‐truncated UNC5C intracellular domain in human *α*‐SNCA mice strongly triggers dopaminergic neuronal loss and motor dysfunction.

## 3 Discussion

In the current study, we show that netrin‐1 reduction in PD patient brains or iPSC‐induced human neurons triggers AEP activation, which robustly cleaves UNC5C at both N467 and N547 residues, augmenting TH‐positive dopaminergic neuronal cell death. Moreover, we also find netrin‐1 is age‐dependently repressed in the brains of *α*‐SNCA transgenic mice, associated with prominent AEP activation and UNC5C fragmentation and dopaminergic neuronal loss. In alignment with these findings, we observe that human *α*‐Syn N103 truncation by AEP is highly escalated in PD brains and aged human *α*‐SNCA transgenic mouse brains, correlated with robust p‐*α*‐Syn S129. Antibodies specific to S129‐phosphorylated *α*‐Syn prominently stain LBs in synucleinopathies.^[^ ^22^ ^]^ S129 phosphorylation, which occurs minimally under physiological circumstances, occurs in the pathologically formed LBs.^[^ ^23^ ^]^ Moreover, co‐staining with ThS/p‐S129 also supports a conclusion that accumulated *α*‐Syn is aggregated into *β*‐sheet, which is recognized by ThS staining (Figure 5). Our previous study shows that human *α*‐Syn is robustly cleaved by active AEP at N103, augmenting *α*‐Syn aggregation.^[^ ^6^ ^]^ Nevertheless, even though the mouse counterpart has no N103 residue, we still observe age‐dependent netrin‐1 reduction and AEP activation, leading to UNC5C N467 fragmentation and TH loss. These observations indicate that UNC5C cleavage by AEP is not dependent on *α*‐Syn N103 truncation. However, because S129 is located after the N103 residue on *α*‐Syn, why is S129 phosphorylation escalated in *α*‐Syn after AEP cleavage? Our recent study shows that truncated *α*‐Syn N103 acts as seeds and forms compact fibrils, inducing endogenous *α*‐Syn monomers to aggregate into Lewy body‐like structures in recipient neurons. Hence, AEP‐truncated *α*‐Syn N103 acts as a pathologic stimulus to provoke soluble and naive *α*‐Syn full‐length proteins to form insoluble inclusions, associated with hyperphosphorylation on S129.^[^ ^8^ ^]^ Theoretically, a protease cleaving a substrate should result in the reduction of the total precursor protein. Nevertheless, active AEP feeds back and activates C/EBP*β* through its substrates‐elicited stress, and C/EBP*β* acts as a major transcription factor for *α*‐SNCA, which subsequently leads to its upregulation^[^ ^24^ ^]^ Therefore, the truncated *α*‐Syn is also replenished by newly synthesized *α*‐Syn due to activation of the C/EBP *β*/AEP pathway.

To investigate the biological consequence of netrin‐1 reduction in the brain, we employed primary neuronal cultures and neutralize the secreted netrin‐1 with its specific antibody or inhibitory proteins. Withdrawal of netrin‐1 elicits robust AEP activation and subsequent UNC5C proteolytic cleavage at N467, which is associated with prominent caspase‐3 activation as well. The UNC5C fragments truncated by AEP are toxic, especially the C‐terminal ones, while the N‐terminal ones bind to netrin‐1 and block its trophic effects, aggravating netrin‐1 deprivation using DCC‐4Fbn and 2F5 antibody. It is interesting to note that expression of UNC5C and its fragments further led to augmented AEP activity and increased cell death upon netrin‐1 withdrawal, indicating that this is a feed‐forward mechanism to amplify the vicious cycle (Figure 4). In SNCA mice, AEP levels are elevated due to oxidative stress‐induced C/EBP *β*, a crucial upstream transcription factor for AEP. Active AEP cleaves numerous substrates including Tau and APP, which are neurotoxic.^[^ ^7^ ^]^ However, we could not detect the aggregated Tau or A*β* in the dopaminergic neurons of SN in our PD mice model. Clearly, future studies to explore more molecular mechanisms involved in this process are needed.

UNC5C and other netrin receptors are also known as dependence receptors, which are responsible for the regulation of neuronal apoptosis, and whether UNC5C plays the role of pro‐ or anti‐apoptotic molecule depends on its binding to netrin‐1.^[^ ^21^, ^25^ ^]^ When netrin‐1 binds to its cognate receptors, these receptors mediate the axon guidance and path‐finding functions during nervous system development.^[^ ^26^ ^]^ In addition, they promote neuronal survival.^[^ ^27^ ^]^ On the other hand, when netrin‐1 is scarce or unavailable, “dependence receptors” induce apoptosis in the absence of the required stimulus, but block cell death in the presence of the required stimulus.^[^ ^14a^ ^]^ Both DCC and rodent UNC5H receptors are cleaved by caspases in their intracellular domain, and this cleavage is a prerequisite for apoptosis induction by the receptors. This cleavage allows the exposure or the release of a pro‐apoptotic domain called addiction/dependence domain.^[^ ^28^ ^]^ In the case of human UNC5A‐D receptors, the caspase cleavage releases a pro‐apoptotic fragment that basically encompasses the whole intracellular domain, which contains the ZU‐5 and DDs. This fragment is sufficient to induce apoptosis when it is myristoylated and overexpressed in immortalized cells.^[^ ^21^ ^]^ The ZU‐5 domain and the DD interact with pro‐apoptotic proteins such as the common neurotrophin receptor, p75, interaction MAGE homolog (NRAGE), and the serine‐threonine death‐associated protein kinase (DAPK).^[^ ^14a^ ^]^ Most recently, we reported that deletion of netrin‐1 in the adult substantia nigra of mice, mimicking the reduction in netrin‐1 levels in PD patient brains, induces DCC cleavage and dopamine neuronal loss, resulting in motor deficits. Remarkably, overexpression of netrin‐1 or brain administration of recombinant netrin‐1 is neuro‐protective and neuro‐restorative in mouse and rat models of PD.^[^ ^12^ ^]^ Therefore, both DCC and UNC5C are implicated in netrin‐1 deficiency‐induced dopaminergic neuronal loss and PD pathologies. Nonetheless, UNC5C, but not DCC or other UNC5 subtypes, is a specific AEP substrate (Figure S2E–H, Supporting Information), albeit they are all substrates of caspase‐3. Though both UNC5C and DCC contribute to netrin‐1 scarcity‐incurred PD pathogenesis, we find that UNC5C is more important in this effect than DCC, because deletion of UNC5C attenuates *α*‐Syn pathologies and decreases dopaminergic neuronal loss more robustly than DCC eradication (Figures 5, 6).

We have previously reported that AEP phosphorylation by the upstream kinase SRPK2 on S226, leads to its translocation from the lysosomes into the cytoplasm^[^ ^29^ ^]^, where it is activated and subsequently cleaves substrates, including *α*‐Syn and UNC5C. Further, caspases cleave the D25 residue on AEP, triggering AEP activation.^[^ ^30^ ^]^ Since both proteases cleave the same substrate UNC5C receptor, to delineate the biological relationship between these two proteases, we employed AEP KO neurons or dominant‐negative C189S mutant (Figure S4, Supporting Information). Compared to potent caspase‐3 activation in wild‐type neurons, netrin‐1 withdrawal weakly activates caspase‐3 in AEP‐null neurons. Consequently, UNC5B and C and DCC cleavage were attenuated. The truncated MW 75 kDa band from UNC5C was completely eradicated but the 50 kDa fragment was only reduced in AEP KO neurons, suggesting that 75 but not 50 kDa might be AEP‐fragmented band. When crippled endogenous AEP with overexpressed C189S mutant, we made the similar observations (Figure S4C, Supporting Information). These observations suggest that AEP somehow mediates caspase activation as well.

Netrins and their receptors DCC and UNC5s are highly expressed in dopaminergic neurons and mediate axon guidance.^[^ ^31^ ^]^ DCC, UNC5C, or netrin‐1 haploinsufficiency leads to increased dopamine content in the medial prefrontal cortex (mPFC) and to resilience against amphetamine‐induced behavioral activation.^[^ ^17^, ^32^ ^]^ Interestingly, reduced expression of UNC5C leads to increased TH expression in the mPFC and to blunted behavioral responses to simulant drugs in adulthood.^[^ ^17a^ ^]^ This finding is consistent with our observations that overexpression of UNC5C reduces TH levels in the SN, indicating dopaminergic neuronal loss (Figures 5, 7). Noticeably, netrin‐1 is substantially reduced in the brains of PD patients and PD mouse models (Figures 1, 2), associated with AEP activation and UNC5C cleavage and dopaminergic neuronal loss. We recapitulated these observations in netrin‐1 f/f mice and *α*‐SNCA mice by selective depletion of netrin‐1. As expected, deletion of UNC5C or DCC partially mitigates these events, indicating that these receptors are responsible for netrin‐1 deletion‐induced biological effects, restoring the motor defects (Figures 5, 6). Remarkably, blockade of UNC5C N467/547 cleavage by AEP provoked by netrin‐1 withdrawal prominently abrogates AEP activation and rescues dopaminergic neuronal loss as compared with UNC5C WT (Figure 5). Consequently, overexpression of AEP‐truncated UNC5C C‐terminal fragment in the SN of *α*‐SNCA transgenic mice strongly augments caspase‐3 and AEP activation, associated with conspicuous dopaminergic neuronal loss, leading to *α*‐Syn aggregation and motor dysfunctions (Figure 7). Thus, AEP cleavage of UNC5C initiates dopaminergic neuronal loss accompanied with evident *α*‐Syn pathologies (Figure 7). Employing viral injection into the SN but not develop transgenic mice to express uncleavable UNC5C N467/547 or UNC5C 468–931 fragment is the limitation of the current study may explain why some of the biochemical or pathological events are only partially blunted.

Together, our current work demonstrates that netrin‐1 reduction in PD brain may induce both caspase‐3 and AEP activation, which robustly cleave DCC and UNC5C that subsequently amplify cell death signals, culminating in dopaminergic neuronal cell death and motor disorders. Because AEP somehow mediates caspase‐3 activation, presumably, inhibition of AEP may strongly block netrin‐1 receptors proteolytic fragmentation, promoting dopaminergic neuronal survival. Given that AEP cuts *α*‐Syn N103 and promotes its aggregation and neurotoxicity,^[^ ^6^ ^]^ conceivably, AEP might be an innovative drug target for treating PD. We have optimized small molecular AEP inhibitor^[^ ^33^, ^34^ ^]^ and improved its IC_50_ potency into sub‐nanomolar range, and this orally bioactive compound will be pushed into the clinical trials for treating PD in the near future.

## 4 Experimental Section

### Mice, Primary Neurons, and Cell Lines

Wild‐type C57BL/6J mice, human *SNCA*–transgenic mice and netrin‐1^flox/flox^ mice were ordered from the Jackson Laboratory (Stock No. 000664, 023837, and 028038, respectively). AEP knockout mice were generated as previously reported^[^ ^7a^ ^]^ All mice were housed in standard conditions at a 12‐h light‐dark cycle and 22  °C with free access to food and water. Animal handling and care were performed following the Emory Medical School guidelines and National Institutes of Health (NIH) animal care guidelines. Sample size was set based on the result from Power and Precision software (Biostat). Mice were assigned to different groups according to a random number table. Investigators were blinded to the group information during the animal experiments. Primary mouse cortical and dopaminergic neurons were cultured as previously described.^[^ ^8^ ^]^ The protocols for these animal experiments were reviewed and approved by the Emory Institutional Animal Care and Use Committee. SH‐SY5Y (ATCC, CRL‐2266) cells were cultured in Advanced DMEM/F12 (Gibco) supplemented with 10% FBS, penicillin (100 U mL^−1^), and streptomycin (100 µg mL^−1^). Cells were incubated in a humidified atmosphere of 5% CO_2_ at 37 °C.

### Human Tissue Samples

Frozen brain samples and tissue sections were from post‐mortem brains of five PD cases and five controls from the Emory Alzheimer's Disease Research Center. Their information is presented in Table S2, Supporting Information. Informed consents were obtained from all subjects. The study was reviewed and approved by the Emory University CND Tissue Committee. PD cases were clinically diagnosed and neuro‐pathologically confirmed. The post‐mortem interval in the PD group was similar to that in the control group.

### Differentiation of Human iPSC‐Derived NPCs into Neurons

Human iPSC‐derived neural progenitor cells (NPCs) were purchased from ATCC (Manassas, USA). Information about the donors is readily available online (https://www.atcc.org/en.aspx). iPSC‐derived NPCs obtained from twp donors: ATCC‐DYS0530 from PD patient, ATCC‐BXS0117 from normal control were used. Neuronal differentiation from NPCs was accomplished by culturing on PLO/Laminin‐coated plates in neuronal differentiation medium, which was composed of DMEM/F12 + Neurobasal Medium (1:1) supplemented with N2, B27, BDNF (20 ng mL^−1^), GDNF (20 ng mL^−1^), NT3 (10 ng mL^−1^), IGF (10 ng mL^−1^), ascorbic acid (200 µm) (all from Stemcell Technologies), and dbcAMP (100 nm) (Sigma Aldrich). The neurons generated from the iPSC differentiation were both NeuN and MAP2 positive as previously described.^[^ ^24^ ^]^

### Antibodies and Reagents

Antibodies to the following targets were used: anti‐GST‐horseradish peroxidase (Sigma Aldrich, A7340), anti‐HA‐horseradish peroxidase (Santa Cruz Biotechnology, sc‐7392), anti‐GFP (Santa Cruz Biotechnology, sc‐9996), anti‐UNC5C 812–823 (Abcam, ab106949), anti‐UNC5C 481–550 (Santa Cruz Biotechnology, sc‐135077), anti‐*β*‐actin (Abcam, ab8227), anti‐AEP antibody clone 11B7 (gift from Dr. Colin Watts, University of Dundee), anti‐active AEP antibody (Cell signaling technology, #93627), anti‐Caspase‐3 (Cell signaling technology, #9662), anti‐cleaved caspase‐3 (Cell signaling technology, #9661), anti‐netrin‐1 (Abcam, ab126729), anti‐UNC5C N467 (Covance),anti‐UNC5C C468 (Covance), anti‐UNC5C N547 (Covance), anti‐DCC (Santa Cruz Biotechnology, sc‐6535), anti‐Protein A/G PLUS‐Agarose (Santa Cruz Biotechnology, sc‐200), *α*‐synuclein (Santa Cruz, sc‐514908), *α*‐synuclein clone 42 (BD, 610787), TH (Santa Cruz, sc‐25269), *α*‐synuclein phospho‐Ser129 (LifeSpan BioSciences, LS‐C380861), anti‐aggregated a‐synuclein antibody (Millipore, MABN389), anti‐*α*‐syn N103 (Ye Lab), anti‐APP N585 (Ye Lab), anti‐APP C586 (Ye Lab), anti‐flag (Sigma Aldrich, F3165), anti‐C/EBP*β* C19 (Santa Cruz, sc‐150), and anti‐p‐C/EBP*β* T188/T235 (CST, #3084).

Reagents: AEP substrate Z‐Ala‐Ala‐Asn‐AMC (Bachem, 4033201), DAPI (Sigma Aldrich, D9542), Mouse and Rabbit Specific HRP/DAB IHC Detection Kit (Abcam, ab236466), CytoTox 96 non‐radioactive cytotoxicity assay (Promega, G1780), 2F5 (AdipoGen, AG‐27B‐0018PF‐C100), DCC‐4Fbn (Ye Lab), UNC5C 459–467 peptide antigen (Covance), Recombinant Mouse Netrin‐1 Protein (R&D systems, 1109‐N1/CF), Phusion High‐Fidelity PCR Kit (NEB, E0553L), In Situ Cell Death Detection Kit, TMR red (Roche, 12156792910), QuikChange Lightning Site‐Directed Mutagenesis Kit (Agilent, 210519), Lipofectamine 3000 (Invitrogen, L3000008), and Recombinant Legumain Protein (rAEP, Sino Biological, 50051‐M07H). All other chemicals not included above were purchased from Sigma Aldrich.

### Plasmids and Viral Vector

HA‐UNC5C and flag‐DCC plasmids were provided by Patrick Mehlen's Lab^[^ ^35^ ^]^ Flag‐UNC5A‐C plasmids were ordered from Addgene. The mGST‐UNC5C plasmids and truncated GFP‐UNC5C plasmids were recombined using Phusion High‐Fidelity PCR Kit. A synonymous mutation (G579T) was performed before GFP tag‐plasmid construction as there was a restriction site on UNC5C template. All the mutations for UNC5C were introduced using site‐directed mutagenesis kit (Agilent Technologies). The primers sequences are presented in Table S1, Supporting Information. The toxicity of full‐length, mutated, or truncated UNC5C was confirmed in SH‐SY5Y cells using Lipofectamine 3000 (Invitrogen) for the transfection. Lentivirus (LV) package for full‐length, truncated, and site‐mutated UNC5C were prepared by Viral vector Core at Emory University. All DNA sequencing was conformed in the Eurofins Genomics LLC. Scramble AAV siRNA control virus (sh‐Control, iAAV01502), UNC5C AAV siRNA Pooled Virus (sh‐UNC5C, 491830940212), DCC AAV siRNA Pooled Virus (sh‐DCC, 177170940212), and netrin‐1 AAV siRNA Pooled Virus (sh‐Netrin‐1, 322460940212) were purchased from ABM company. AAV‐Cre‐GFP (Cre, 7019), AAV‐GFP (Control, 7008) were ordered from Vector Biolabs. Viral titer was 1 × 10^12^ vg mL^−1^ for AAV virus constructions and 1 × 10^9^ vg mL^−1^ for lentiviral constructions. AAV‐AEP C189S virus was provided by Dr. Fredric Manfredsson's Lab.

### In Vitro Cleavage Assay

To explore UNC5C cleavage by AEP in vitro, HEK293 cells were transfected with mGST‐UNC5C or HA‐UNC5C WT or mutated plasmids by the calcium phosphate precipitation method. 48 h after transfection, the cells were washed with PBS, collected and lysed in buffer (5 mm DTT, 50 mm sodium citrate, 0.1% CHAPS, and pH 5.5, 0.5% Triton X‐100) for 5 min, and then centrifuged at 15 000 g at 4 °C for 15 min. The resulting supernatant was incubated with mouse brain lysates or combined with rAEP (5 µg mL^−1^) for 30 min under pH 7.4 or 6.0 at 37 °C. rAEP was first activated before use by incubation in activation buffer (0.1 m NaOAc, 0.1 m NaCl, pH 4.5) for 4 h at 37  °C. To detect the effect of AEP inhibitor on UNC5C cleavage by AEP, AENK peptide, and the inactive control AEQK were used. The cleavage assay for flag‐UNC5A‐C and DCC plasmids followed the same method.

### AEP/*δ*‐Secretase Activity Assay

Cell lysates or brain tissue homogenates were incubated in 200 µL assay buffer (60 mm Na2HPO4, 20 mm citric acid, 1 mm EDTA, 0.1% CHAPS, and 1 mm DTT, pH 6.0) containing 20 µm AEP substrate Z‐Ala‐Ala‐Asn‐AMC. AMC released from substrate cleavage was quantified by measuring at 460 nm in a fluorescence plate reader at 37  °C for 1 h in kinetic mode. The activity of AEP was expressed as the reading at 1 h minus the baseline reading.

### Generation of AEP‐Derived UNC5C Fragment Antibodies

The anti‐UNC5C N467, anti‐UNC5C C468, and anti‐UNC5C N547 antibodies were developed by immunizing rabbits with the following peptides: Ac‐CVSDKIPMTN‐OH (anti‐UNC5C N467), H2N‐SPILDPLPNC‐amide (anti‐UNC5C C468), and Ac‐CLGGHLIIPN‐OH (anti‐UNC5C N547), respectively. The antiserum was pooled, and the titer against the immunizing peptides was determined by ELISA. The maximal dilution showing a positive response using chromogenic substrate for HRP was >1:750 000. The immunoactivity of the antiserum was further verified by IHC and western blot. Then the antiserum was purified with Affi‐Gel 10.

### Stereotaxic Injection of the Viral Vector

3 month‐old netrin‐1^flox/flox^ mice and human SNCA–transgenic mice were anesthetized with isoflurane (Piramal Healthcare). Meloxicam (2 mg kg^−1^) was injected subcutaneously for analgesics (Loxicom, Norbrook). Bilateral intranigral injection of 2 µL viral vectors was performed stereotaxically at coordinates mediolateral −1.2 mm and anteroposterior −3.1 mm relative to the bregma, and dorsoventral −4.3 mm from the dura surface, using 10‐µL glass syringes with a fixed needle at a rate of 0.5 µL min^−1^. The needle stayed in place for 5 min before it was removed slowly (not less than 2 min). The mice were placed on heating pad until they began to recover from the surgery.

### Western Blot Analysis

The human tissue and mouse brain tissue samples were lysed in lysis buffer (40 mm NaCl, 1 mm EDTA, 50 mm Tris, pH 7.4, 0.5% Triton X‐100, 50 mm NaF, 1.5 mm Na_3_VO_4_, 10 mm sodium pyrophosphate, and 10 mm sodium *β*‐glycerophosphate, supplemented with protease inhibitors cocktail) on ice, and centrifuged at 16 000 g at 4 °C for 20 min. The samples for analyzing the level of soluble and insoluble *α*‐synuclein were prepared as previously described.^[^ ^36^ ^]^ The supernatants from Triton X‐100 cell lysates were collected as soluble fractions. The resultant pellets were further lysed in the solution containing 2% SDS and 8 m urea, sonicated at 30 W for 1 s 10 times, and centrifuged at 100 000 × g for 30 min. Resultant supernatants were collected as insoluble fractions. The supernatants were boiled in 1 × SDS loading buffer. After SDS‐PAGE, the samples were transferred to nitrocellulose membrane. Western blot analysis was carried out with various antibodies. Images were cropped for presentation.

### Immunostaining

Paraffin‐embedded human brain sections and Free‐floating mouse brain sections were detected by immunostaining with a variety of antibodies. Paraffin‐embedded human brain sections were dewaxed with xylene, treated with gradient alcohol and washed with PBS. Then these sections were treated with 3% H_2_O_2_ at room temperature for 10 min, washed three times with PBS. After antigen retrieval in boiling sodium citrate buffer, the sections were blocked in 1% BSA containing 0.3% Triton X‐100 for 60 min and incubated with primary antibodies at 4 °C overnight. The signal for immunochemistry was developed using the Histostain‐SP kit. The signal for IF was developed using secondary antibody, Alexa Fluor 594, Alexa Fluor 488 or Cyanine5. For Thioflavin‐S staining, the sections were incubated for 8 min with 0.0125% Thioflavin‐S in 50% ethanol, washed with 50% ethanol and distilled water. The sections were covered with a glass cover along with using mounting solution and examined under an optical microscope or fluorescence microscope. The researchers who performed immunostaining and analysis were blinded to the group allocations.

### Cytokine ELISA

Mouse SN samples were lysed in lysis buffer (40 mm NaCl, 50 mm Tris, pH 7.4, 1 mm EDTA, 1.5 mm Na3VO4, 50 mm NaF, 10 mm sodium pyrophosphate, 0.5% Triton X‐100, and 10 mm sodium *β*‐glycerophosphate, supplemented with protease inhibitors cocktail), and centrifuged for 20 min at 15 000 rpm (4  °C). The isolated supernatant part for cytokine (IL1*β*, IL‐6, and TNF‐*α*) levels analysis use ELISA kits (ThermoFisher, IL‐1*β* Mouse Uncoated ELISA Kit with Plates, Cat # 88‐7013‐22; IL‐6 Mouse Uncoated ELISA Kit with Plates, Cat # 88‐7064‐22; TNF‐*α* Mouse Uncoated ELISA Kit with Plates, and Cat # 88‐7324‐22) according to the manual.

### Quantification and Statistical Analysis

ImageJ software was used to quantify the branches of primary dopaminergic neurons^[^ ^37^ ^]^, Western blots, IHC, and IF experiments. GraphPad Prism 8 software was used for analyses of results. Statistical analysis was performed using Student's *t*‐test for two‐group comparison, one‐way ANOVA followed by Tukey's multiple‐comparisons test for more than two groups, or two‐way ANOVA followed by Bonferroni's post hoc test for repeated measures. The data are presented as mean ± s.e.m. Differences with *p* value less than 0.05 were considered significant.

## Conflict of Interest

The authors declare no conflict of interest.

## Author Contributions

K.Y. conceived the project, designed the experiments, analyzed the data, and wrote the manuscript. G.C., E.H.A., S.S.K. and Y.X. designed and performed most of the experiments. X.L. prepared primary neurons and assessed with animal experiments. Z.Z. assisted with data analysis and interpretation and critically read the manuscript.

## Supporting information

Supporting Information

## Data Availability

All data needed to support the findings of this study are present in the paper and the Supporting Information.
