# Supplementary material for: UNC5C Receptor Proteolytic Cleavage by Active AEP Promotes Dopaminergic Neuronal Degeneration in Parkinson's Disease
Source: Adv Sci (Weinh). 2022 Jan 12;9(7):2103396. doi: 10.1002/advs.202103396 (PMC8895126; doi:10.1002/advs.202103396)
Supplement: Supplementary file 1 — Supporting Information [file ADVS-9-2103396-s001.pdf]

ADVANCED SCIENCE
Open Access

Supporting Information

for *Adv. Sci.*, DOI: 10.1002/advs.202103396

UNC5C Receptor Proteolytic Cleavage by Active AEP Promotes Dopaminergic Neuronal Degeneration in Parkinson's Disease

Guiqin Chen [1, 2], Eun Hee Ahn[1], Seong Su Kang [1], Yiyuan Xia[1], Xia Liu [1], Zhaohui Zhang [2] and Keqiang Ye[1, 3, 4,]*

Supplementary Figure 1

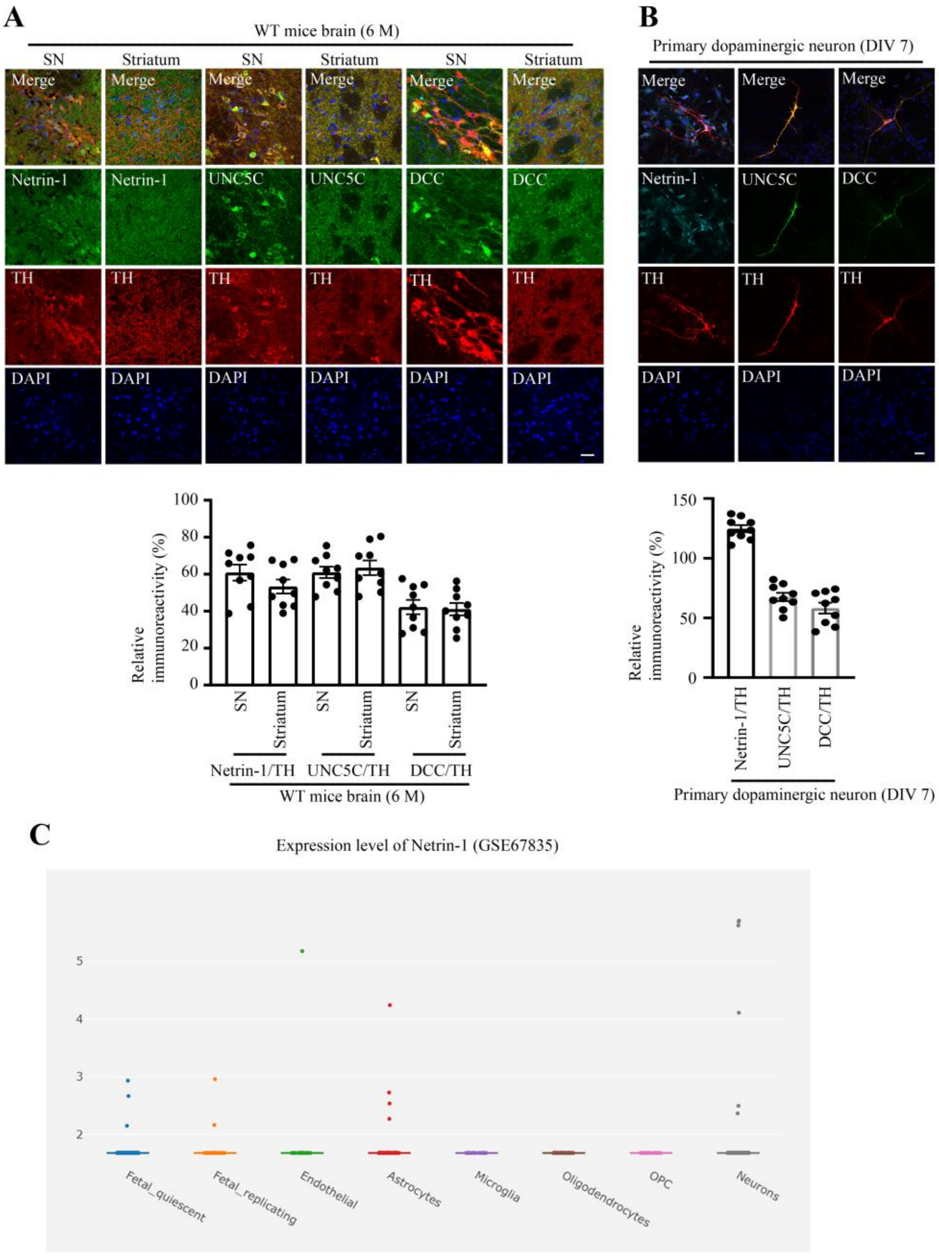

Supplementary Figure 1. Netrin-1 and its receptors, UNC5C and DCC, are expressed in dopaminergic neurons

(A) Immunofluorescence co-localization analysis was performed to detect the expression of Netrin-1, UNC5C and DCC in the SN and striatum of 6 months old mice brains. TH was used as

the marker of dopaminergic neuron. Scale bar, 30 μm. Data are mean ± s.e.m.; n = 3 mice per group. **(B)** Netrin-1, UNC5C and DCC expression were shown in primary dopaminergic neurons at days in vitro (DIV) 7 by immunofluorescence staining. Scale bar, 30 μm. Data are mean ± s.e.m.; n = 3 slices per group. **(C)** Netrin-1 expression level detected by a survey of human brain transcriptome diversity at the single cell level (GSE67835). This image is generated from the AlzData database.

Supplementary Figure 2

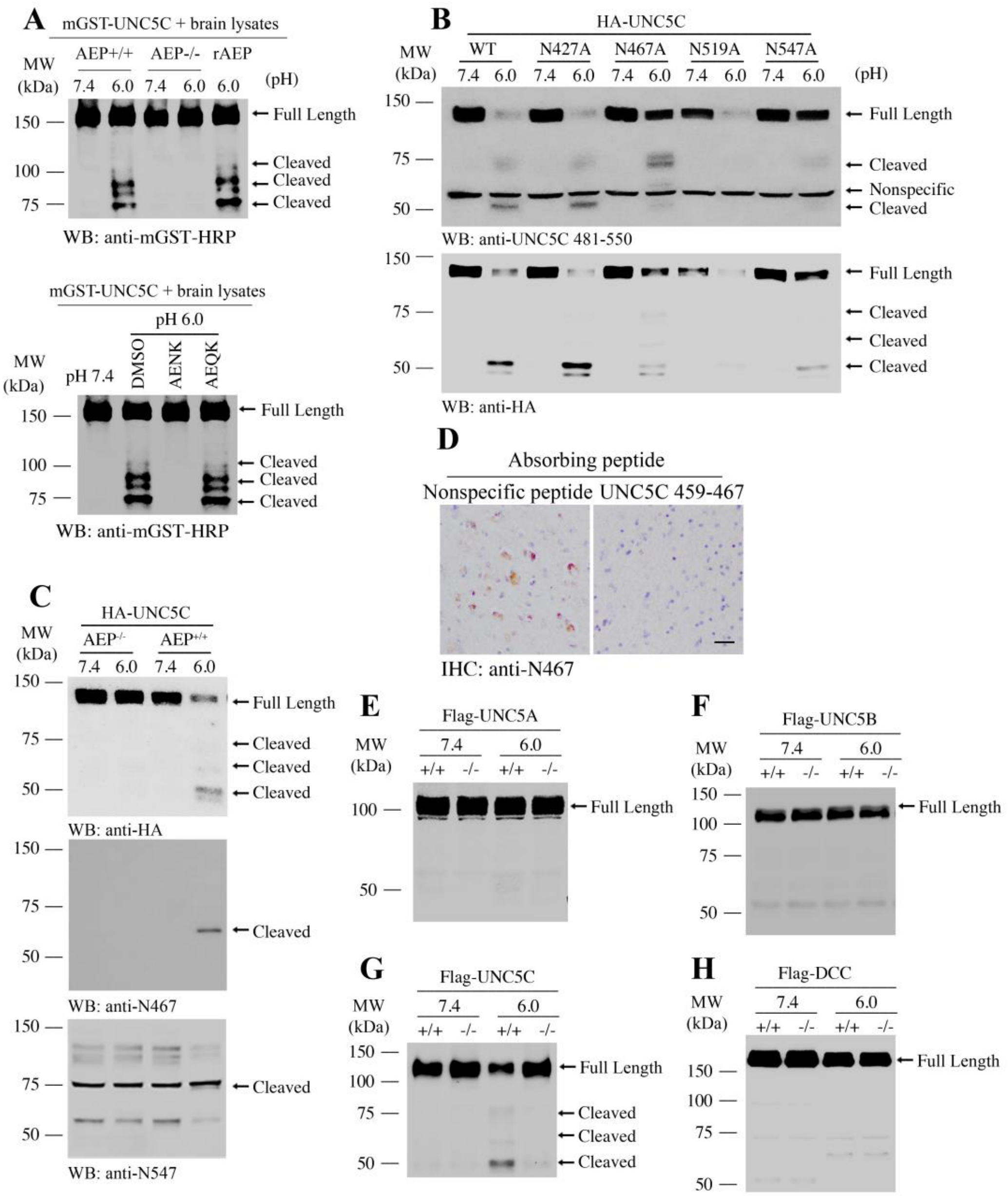

Supplementary Figure 2. N467 and N547 are the main residues of UNC5C receptor cleaved by AEP *in vitro*

(A) AEP cleaved UNC5C receptor *in vitro* and inhibition of AEP blocked the cleavage. Mammalian GST-tagged UNC5C construct was transfected and recombinant proteins were isolated and subjected to *in vitro* cleavage in AEP +/+ and -/- brain lysates. Re-introduction of rAEP into AEP -/- brain lysates restored UNC5C cleavage (upper panel). mGST-UNC5C was incubated in wild-type brain lysates under pH 6.0 in the presence of AENK or AEQK peptide. Immunoblotting showed blockade of AEP by its peptide inhibitor antagonized UNC5C cleavage (lower panel). **(B)** Mutation of UNC5C at N467 or N547 decreased UNC5C receptor cleavage by AEP. HEK 293 cells were transfected with HA-UNC5C WT, N427A, N467A, N519A or N547A plasmids. In 36 hours, cells were collected and lysed for cleavage assay. HA-UNC5C WT and its mutated ones were incubated with rAEP recombinant proteins at pH 7.4 or 6.0 for 1 hour, respectively. The cleavage patterns were detected with anti-UNC5C and anti-HA antibodies using Western blot (WB). **(C).** Anti-N467 antibody specifically recognized UNC5C N467 fragment. HA-UNC5C WT was incubated with brain lysates derived from wild-type ($AEP^{+/+}$) or AEP knockout ($AEP^{-/-}$) mice at pH 7.4 or 6.0, respectively. Western blot (WB) showed that UNC5C was cleaved at pH 6.0 in $AEP^{+/+}$ brain lysates when AEP was activated. (**D**). Confirmation of the specificity of anti-N467 antibody. The anti-N467 antibody was preincubated with a peptide (UNC5C 459–467) or nonspecific peptide before immunohistochemistry. The signal was blocked by UNC5C 459–467 peptide but not the nonspecific one. Scale bar, 30 μm. **(E-H)** Flag-UNC5A-C and DCC proteins were incubated with mice brain lysates (wild-type, +/+; AEP knockout, -/-). WB showed that Flag-UNC5A-B and DCC were not obviously cleaved by δ-secretase, but Flag-UNC5C was selectively cleaved by δ-secretase. ◄, full length; ←, cleaved.

Supplementary Figure 3

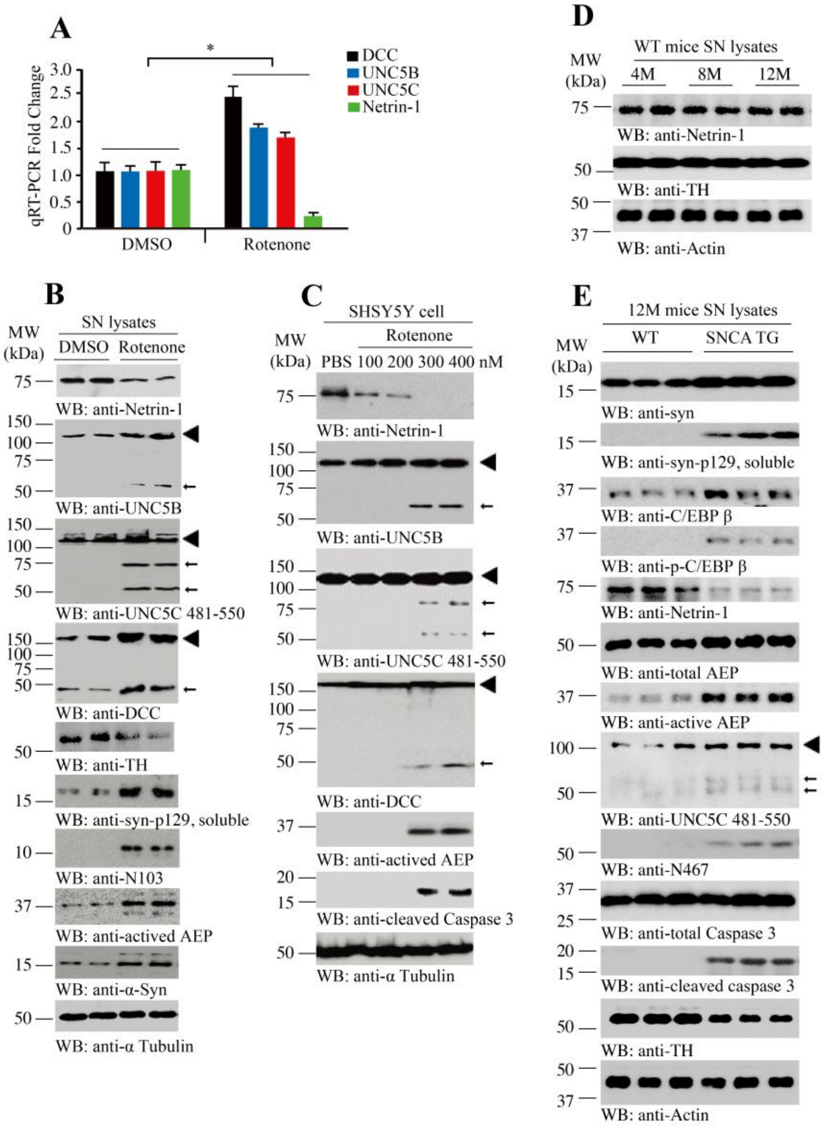

Supplementary Figure 3. Netrin-1 withdrawal elicits AEP activation and UNC5C cleavage

(A) Rotenone treatment affected Netrin-1 and its receptors expression in human α-SNCA transgenic mice. The control group mice received DMSO and the PD group received the

rotenone (2.5 mg/kg) for 3 months via oral gavages injection method. The mRNA levels in SN were analyzed by real-time PCR. DCC (black), UNC5B (blue), UNC5C (red) and netrin1 (green). All real-time PCR experiments were repeated at least 3 times. Error bars represent the mean ± s.e.m.. Statistical significance was determined using a two-way ANOVA followed by post hoc Bonferroni test for multiple group comparisons. P**< 0.01. **(B)** Immunoblot analysis representative images showing Netrin-1, UNC5B, UNC5C, DCC, TH, pS129, α-N103, α-Syn FL and AEP expression levels in SN of above mice. (**C)** Rotenone treatment represses netrin-1 and elicits active AEP and UNC5C cleavage in SH-SY5Y cells. Dopaminergic cells SH-SY5Y were treated with different doses of rotenone for 2 days, followed by immunoblotting analysis with indicated antibodies. (**D)** Netrin-1 and TH levels detected by WB show no significant difference in the SN of WT mice at the ages of 4, 8 and 12 months (4M, 8M, 12M). **(E)** C/EBP β is highly activated in human α-SNCA transgenic mice (α-SNCA TG) compared to age-matched WT mice (12-month old), accompanied by netrin-1 reduction, UNC5C cleavage, AEP and caspase 3 activation. ◄, full length; ←, cleaved.

Supplementary Figure 4

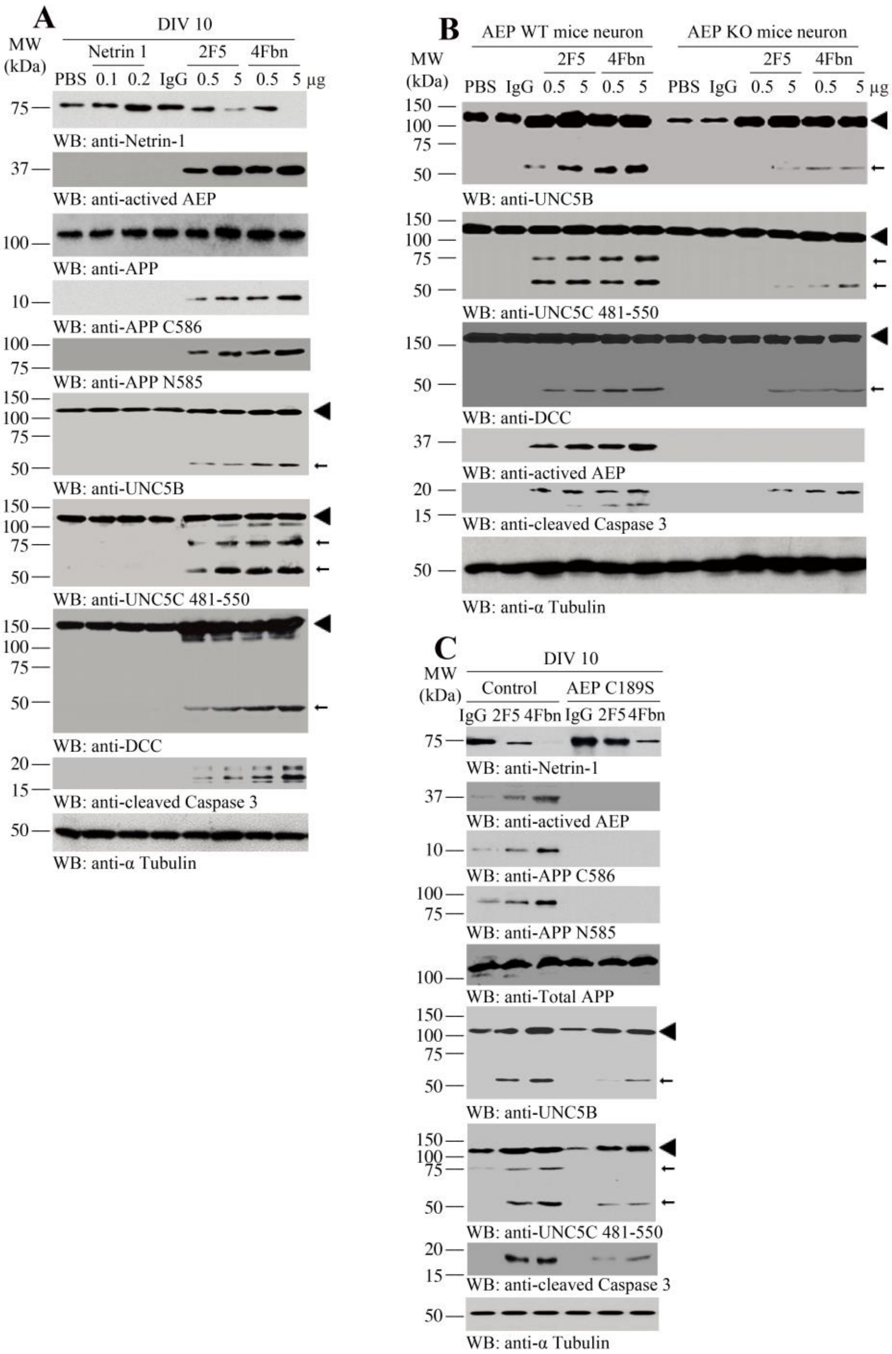

Supplementary Figure 4. AEP enzyme cleaves UNC5C receptor in primary neurons

(A) Neutralization of netrin-1 in primary neuronal cultures activated AEP, inducing UNC5C proteolytic cleavage. DIV 10 primary neuronal cultures were treated with control mIgG or anti-netrin-1 2F5 or recombinant protein DCC-4Fbn to neutralize netrin proteins secreted in the media. The neuronal lysates were prepared and analyzed by immunoblotting with indicated antibodies. **(B)** Knockout of AEP robustly attenuated netrin-1 withdrawal-induced UNC5C cleavage. Primary mouse cortical neurons were cultured from WT and AEP –knockout neonatal pups. In 10 days (DIV 10), the primary cultures were treated with indicated antibody or recombinant proteins to neutralize the secreted netrins in the media. The neuronal lysates were analyzed with indicated antibodies via immunoblotting. (**C)** Overexpression of inactive AEP C189S in primary neuronal cultures suppressed netrin-1 withdrawal-elicited UNC5C cleavage. DIV 10 primary neuronal cultures were infected with AAV-AEP C189S, followed by addition of control mIgG or anti-netrin-1 2F5 or recombinant protein DCC-4Fbn to neutralize netrin proteins secreted in the media. The neuronal lysates were prepared and analyzed by immunoblotting with indicated antibodies.

Supplementary Figure 5

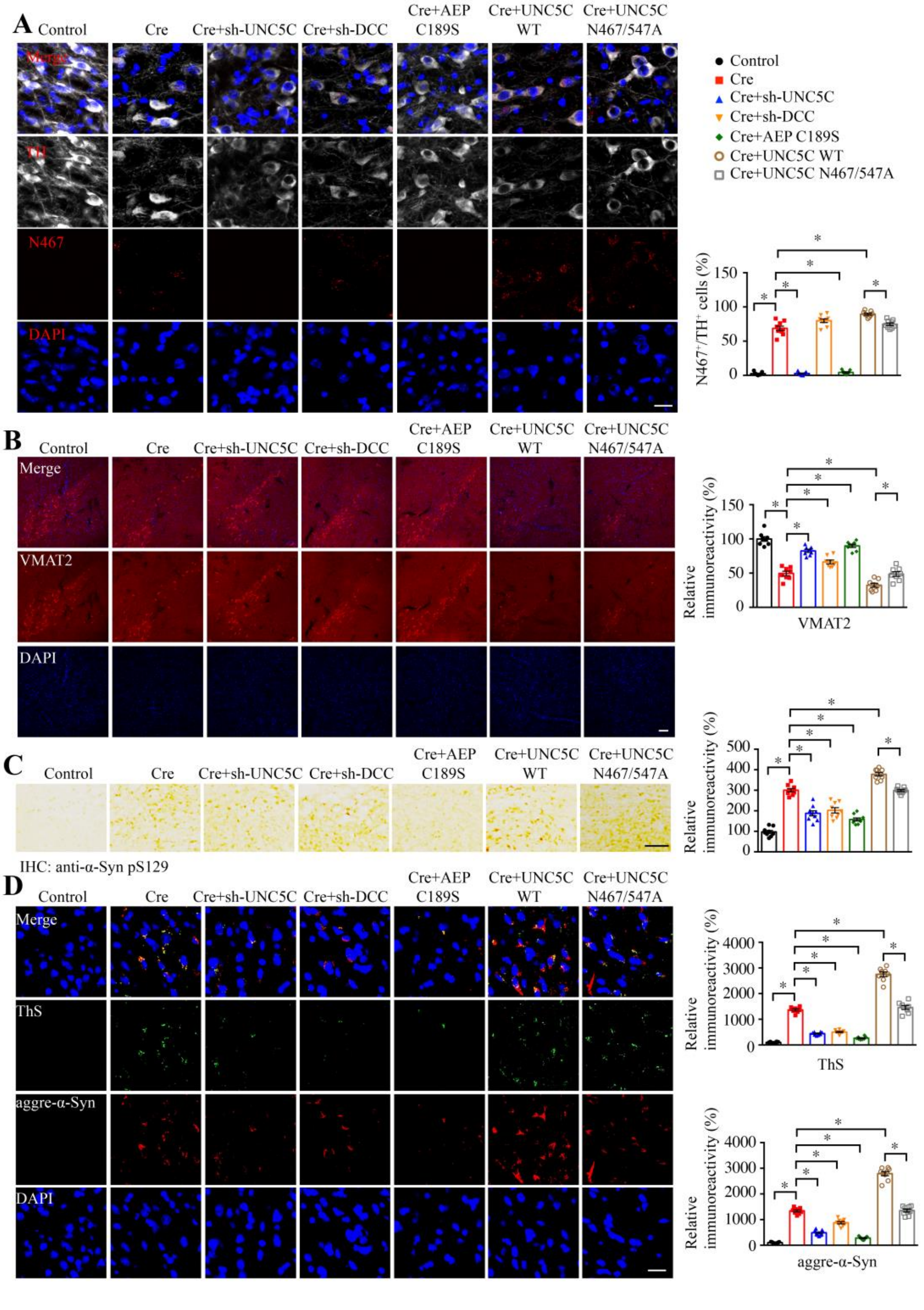

Supplementary Figure 5. Netrin-1 deprivation in substantia nigra of Netrin f/f mice induces AEP activation and α-Syn aggregation

(A & B) Blockade of UNC5C cleavage by AEP reduced dopaminergic neuron loss in the SN of netrin f/f mice. **(A)** Immunofluorescent signals of anti-TH (white) and anti-N467 (red) were detected. The nuclei were stained with DAPI. Quantification showing the percentage of $N467^+$ cells co-expressed with TH^+ cells. Scale bar, 20 μm. **(B)** Immunofluorescent signals of anti-VMAT2 was detected to further verify dopaminergic neuron loss. The nuclei were stained with DAPI. Scale bar, 60 μm. **(C)** Immunohistochemistry staining was performed to analyze α-synuclein pS129 (α-syn pS129) expression in the SN of above animals. Scale bar, 500 μm. **(D)** Blockade of UNC5C cleavage by AEP attenuated Lewy body-like structures in the SN in netrin f/f mice. Immunofluorescent signals of Thioflavin S (ThS, green) and anti-aggregated α-synuclein (aggre-α-syn, red) were detected. The nuclei were stained with DAPI. Scale bar, 20 μm. Data are mean ± s.e.m. (n = 3 mice per group). *$P < 0.05$ by one-way ANOVA followed by Tukey's multiple-comparisons test.

Supplementary Figure 6

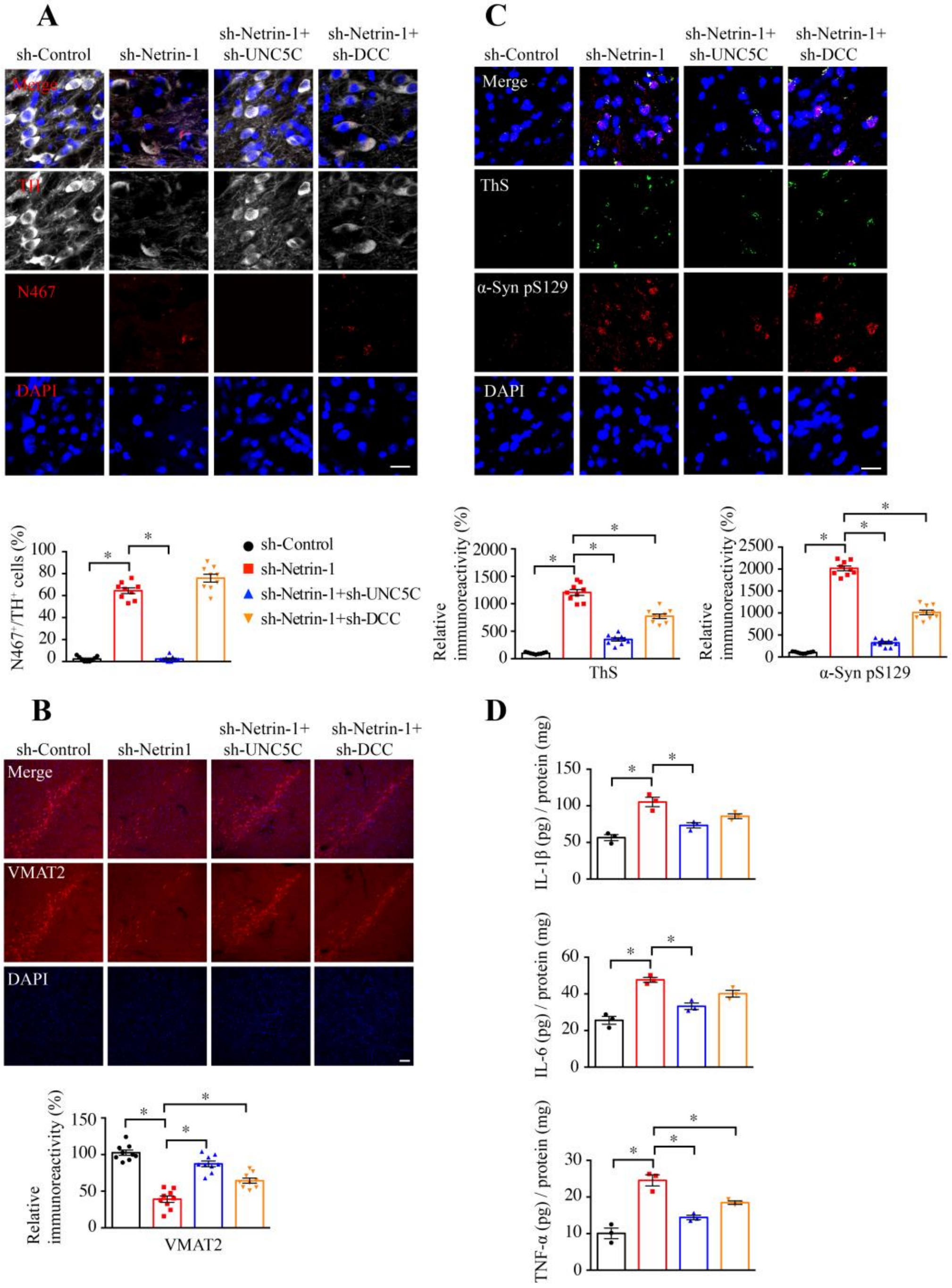

Supplementary Figure 6. Netrin-1 deprivation in substantia nigra of SNCA transgenic mice induces AEP activation and α-Syn aggregation

(A & B) Knockdown of UNC5C strongly reduced netrin-1 depletion-elicited UNC5C cleavage by AEP and dopaminergic neuron loss. **(A)** Immunofluorescent signals of anti-TH (white) and anti-N467 (red) were detected in SN. The nuclei were stained with DAPI. Quantification showing the percentage of $N467^+$ cells co-expressed with TH^+ cells. Scale bar, 20 μm. **(B)** Immunofluorescent signals of anti-VMAT2 were detected to further verify dopaminergic neuron loss. The nuclei were stained with DAPI. Scale bar, 60 μm. **(C)** Immunofluorescent signals of Thioflavin S (ThS, green) and anti-α-synuclein p-S129 (α-syn p-S129, red) were detected in SN. The nuclei were stained with DAPI. Quantification of ThS and α-syn p-S129 relative expression levels. Scale bar, 20 μm. **(D)** Cytokine (IL-1β, IL-6, TNFα) ELISA analysis showed cytokines levels in the above samples. Data are mean ± s.e.m. (n = 3 mice per group). $*P < 0.05$ by one-way ANOVA followed by Tukey's multiple-comparisons test.

Supplementary Figure 7

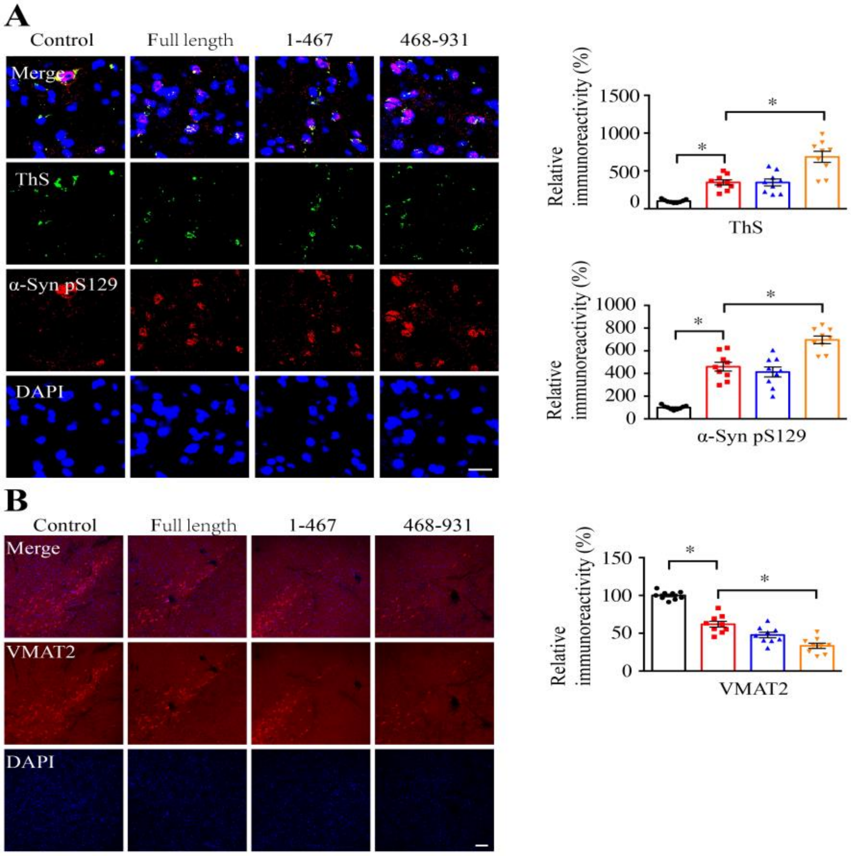

Supplementary Figure 7. Overexpression of AEP-cleaved UNC5C fragments enhances α-Syn aggregation and dopaminergic neuron loss in SNCA transgenic mice

(A) AEP-truncated UNC5C fragments escalated α-Syn phosphorylation and aggregation. Immunofluorescent signals of Thioflavin S (ThS, green) and anti-α-synuclein p-S129 (α-syn p-S129, red) were detected in SN. The nuclei were stained with DAPI. Scale bar, 20 μm. Quantification of ThS and p-α-syn pS129 relative expression levels. **(B)** Immunofluorescent staining of VMAT2 was used to verify the dopaminergic neuron loss. The nuclei were stained with DAPI. Scale bar, 60 μm. Data are mean ± s.e.m. (n = 3 mice per group). *$P < 0.05$ by one-way ANOVA followed by Tukey's multiple-comparisons test.

Supplementary Table 1 Primers for construct plasmids

Primer Name	Primer Sequence (5' to 3')
mouse UNC5C Sal1 F	ACT GTCGAC C ATG AGG AAA GGT CTG AGG GCG
mouse UNC5C Not1 R931	ACT GC GGC CGC TCA ATA CTG TCC TTC TGC TGC
mouse UNC5C N467A F	CAGACAAAATCCCAATGACCGCCTCTCCAATTCTGGACCCAC
mouse UNC5C N467A R	GTGGGTCCAGAATTGGAGAGGCGGTCATTGGGATTTTGTCTG
mouse UNC5C N547A F	GGGTCACCTCATCATTCCTGCTTCAGGAGTAAGCTTGCTG
mouse UNC5C N547A R	CAGCAAGCTTACTCCTGAAGCAGGAATGATGAGGTGACCC
mouse UNC5C G579T F	GGCCACCTGAAGGTATCCCAGTGGCTGAGG
mouse UNC5C G579T R	CCTCAGCCACTGGGATACCTTCAGGTGGCC
mouse UNC5C EcoR1 F	CGC GAATTC ATG AGG AAA GGT CTG AGG GCG
mouse UNC5C BamH1 R931	ACC GGATCC TT ATA CTG TCC TTC TGC TGC CAA GGA CAC
mouse Unc5c1-467BamH1R	ACC GGATCC TT GTT GGT CAT TGG GAT TTT GTC TGA GAC
mouse Unc5c1-547BamH1R	ACC GGATCC TT ATT AGG AAT GAT GAG GTG ACC CCC AAG
mouse Unc5c468-931EcoR1F	CGC GAATTC ATG TCT CCA ATT CTG GAC CCA CTA CCC
mouse Unc5c548-931EcoR1F	CGC GAATTC ATG TCA GGA GTA AGC TTG CTG ATT CCC

Supplementary Table 2 Human Brain Samples Information

Cases	Age at Onset	Age at Death	Sex
Control	X	57	male
Control	X	94	male
Control	X	91	female
Control	X	61	female
Control	X	70	male
PD	74	84	female
PD	67	74	male
PD	77	90	female
PD	52	58	male
PD	48	69	male
